# HER2DX genomic test in HER2-positive/hormone receptor-positive breast cancer treated with neoadjuvant trastuzumab and pertuzumab: A correlative analysis from the PerELISA trial

**DOI:** 10.1016/j.ebiom.2022.104320

**Published:** 2022-10-29

**Authors:** Valentina Guarneri, Fara Brasó-Maristany, Maria Vittoria Dieci, Gaia Griguolo, Laia Paré, Mercedes Marín-Aguilera, Federica Miglietta, Michele Bottosso, Carlo Alberto Giorgi, Paula Blasco, Oleguer Castillo, Patricia Galván, Ana Vivancos, Patricia Villagrasa, Joel S. Parker, Charles M. Perou, PierFranco Conte, Aleix Prat

**Affiliations:** a Department of Surgery, Oncology and Gastroenterology, University of Padova, Padova, Italy; b Istituto Oncologico Veneto, IRCCS, Padova, Italy; c Translational Genomics and Targeted Therapies in Solid Tumors, August Pi i Sunyer Biomedical Research Institute (IDIBAPS), Barcelona, Spain; d Reveal Genomics, Barcelona, Spain; e Cancer Genomics Group, Vall d'Hebron Institute of Oncology (VHIO), Barcelona, Spain; f Lineberger Comprehensive Cancer Center, University of North Carolina, Chapel Hill, USA; g Department of Genetics, University of North Carolina, Chapel Hill, USA; h Department of Medical Oncology, Hospital Clinic of Barcelona, Spain; i Department of Medicine, University of Barcelona, Barcelona, Spain; j Institute of Oncology (IOB)-Hospital Quirónsalud, Barcelona, Spain

**Keywords:** HER2-positive, Breast cancer, HER2DX, Biomarker, Gene expression, Trastuzumab, Pertuzumab

## Abstract

**Background:**

HER2DX is a prognostic and predictive assay in early-stage HER2-positive breast cancer based on clinical features and the expression of 4 gene signatures (immune, proliferation, luminal differentiation and HER2 amplicon), including ERBB2 mRNA levels. Here, we evaluated the ability of HER2DX to predict efficacy of a de-escalated, chemotherapy-free neoadjuvant regimen in HER2-positive/hormone receptor-positive breast cancer.

**Methods:**

HER2DX was evaluated on pre-treatment tumour samples from the PerELISA phase II study focused on postmenopausal patients with operable HER2-positive/hormone receptor-positive breast cancer. Patients received 2-weeks of letrozole, and then underwent a re-biopsy for Ki67 evaluation. Patients with endocrine therapy sensitive disease (ESD) (i.e., >20.0% Ki67 relative reduction at week 2) continued letrozole and 5 cycles of trastuzumab and pertuzumab. Primary aim was to test the ability of HER2DX risk-score, HER2DX pCR score and HER2DX ERBB2 mRNA score (as continuous variables and group categories) to predict pathological complete response (pCR) in patients with ESD. Logistic regression and receiver–operator curve (ROC) analysis assessed associations of HER2DX scores with pCR and ESD.

**Findings:**

HER2DX was evaluated in 55 patients (86.0%) enrolled in PerELISA and 40 patients (73.0%) had ESD. The pCR rate in patients with ESD was 22.5% (9/40). In this group, HER2DX pCR score and HER2DX ERBB2 mRNA score were significantly associated with pCR (p = 0.008 and p = 0.003, univariate logistic regression model; area under ROC [AUC] = 0.803 and 0.896). The pCR rate in low, medium, and high HER2DX pCR score groups was 7.7% (2/26), 46.2% (6/13) and 100.0% (1/1), respectively. The pCR rate in low, medium, and high HER2DX ERBB2 score groups was 0.0% (0/12), 7.7% (1/13) and 53.3% (8/15), respectively. HER2DX pCR score was also significantly associated with Ki-67 response following 2-weeks of letrozole (p = 0.002, univariate logistic regression model; AUC = 0.775). The rate of ESD in low, medium, and high HER2DX pCR score groups was 89.7% (26/29), 65.0% (13/20) and 16.7% (1/6), respectively.

**Interpretation:**

HER2DX predicts response following neoadjuvant letrozole in combination with dual HER2 blockade with trastuzumab and pertuzumab in early-stage HER2-positive/hormone receptor-positive breast cancer.

**Funding:**

This study received funding from Reveal Genomics.

**Research in context**

**Evidence before this study**

We searched PubMed for clinical trials or studies published in English between January 1, 2010, and August 1, 2022, assessing HER2 inhibition in early-stage breast cancer, with the search terms “HER2-positive”, “early-stage”, “de-escalation”, “biomarker”, “breast cancer”, and “anti-HER2 therapy”. In patients with early-stage HER2-positive breast cancer, clinical guidelines support the use of (neo)adjuvant anti-HER2-based targeting plus chemotherapy for most patients. However, various strategies to de-escalate systemic therapy have been evaluated, such as eliminating the amount, or even the use, of chemotherapy. One of these studies, the PerELISA phase II trial, evaluated a strategy of 5-cycles of neoadjuvant letrozole, trastuzumab and pertuzumab in 44 patients with early-stage HER2-positive/hormone receptor-positive breast cancer with endocrine sensitivity disease, selected on the basis of Ki67 reduction after 2-week letrozole exposure. The study found that 20.5% of patients achieved a pathological complete response (pCR) and that a PAM50 HER2-enriched genomic profile, determined in baseline pre-treatment tumour samples, was found associated with a higher likelihood of achieving a pCR than the other subtypes (46% vs. 14%). Another study, the PHERGain phase II trial, treated 227 patients with early-stage HER2-positive breast cancer with 8-cycles of neoadjuvant trastuzumab and pertuzumab (and endocrine therapy if hormone receptor-positive), and the pCR rate was 37.9%. Despite the successes and limitations of these de-escalation strategies, most patients with early-stage, HER2-positive breast cancer are still treated with chemotherapy today; therefore, there is a need for implementing new tools to help guide the use of cytotoxic therapy in early-stage, HER2-positive breast cancer.

In 2022, we reported the development and clinical validation of HER2DX, a genomic assay that integrates clinical data with genomic data and thus captures tumour features, immune features, and pathology features all in one assay. HER2DX uses the information captured by the assay to predict two different clinical endpoints, namely, long-term survival outcome and likelihood of achieving a pathological complete response (pCR) following neoadjuvant anti-HER2-based therapy. In addition, the HER2DX reports an ERBB2 mRNA score as a continuous variable and with validated cut-offs. To date, however, the value of HER2DX to predict response to dual HER2 blockade with trastuzumab and pertuzumab in the absence of chemotherapy is unknown, as well as the value of HER2DX to predict endocrine sensitivity.

**Added value of this study**

This report shows clinical validation of HER2DX in patients with early-stage HER2-positive/hormone receptor-positive breast cancer treated with trastuzumab, pertuzumab and letrozole. In addition, HER2DX provides more predictive information compared to the PAM50 HER2-enriched subtype.

**Implications of all the available evidence**

The evidence suggests that HER2DX might be able to identify upfront a substantial proportion of patients with early-stage, HER2-positive/hormone receptor-positive breast cancer who might not need chemotherapy if treated with dual HER2 blockade and endocrine therapy. Additional studies will further solidify the clinical utility of HER2DX scores to help de-escalate systemic and/or loco-regional treatments.

## Introduction

In the last 5 years, introduction of anti-HER2 therapies such as pertuzumab, T-DM1 or neratinib has improved survival outcomes in early-stage HER2-positive breast cancer beyond trastuzumab and chemotherapy.^1^^,^^2^ However, substantial clinical and biological heterogeneity exists in HER2-positive disease, which affects patients' prognosis and treatment benefit.^2^, ^3^, ^4^, ^5^ Thus, strategies to optimize therapy and improve quality of life have been recently explored for patients with early-stage HER2-positive breast cancer.

One strategy being pursued is the elimination of chemotherapy in HER2-positive disease.^6^^,^^7^ In this direction, several neoadjuvant trials have explored the combination of 2 anti-HER2 drugs (i.e., dual HER2 blockade) with either trastuzumab-lapatinib or trastuzumab-pertuzumab in the absence of cytotoxic therapy.^8^, ^9^, ^10^, ^11^, ^12^, ^13^, ^14^ Overall, this treatment strategy achieves pCR rates of 20–40% after 4–6 months of therapy.^8^, ^9^, ^10^, ^11^, ^12^, ^13^ However, HER2-positive breast cancer is generally chemosensitive and the rates of pCR in the absence of chemotherapy are lower than in the presence of chemotherapy.^12^^,^^15^ In addition, the impact in survival of dual HER2 blockade without cytotoxic therapy is unclear, and large (neo)adjuvant trials such as PHERGain (NCT03161353) and PHERGain-2 (NCT04733118) are still ongoing. Therefore, most patients with early-stage, HER2-positive breast cancer are treated today with chemotherapy regardless of hormone receptor status.

Implementing new tools to help guide the use of cytotoxic therapy in early-stage, HER2-positive breast cancer is of high interest. Several variables beyond tumour burden have been associated with patients' prognosis and/or treatment response in early-stage, HER2-positive breast cancer. For example, the percentage of stromal tumour-infiltrating lymphocytes,^16^, ^17^, ^18^ hormone receptor status, and the intrinsic molecular subtypes of breast cancer.^15^^,^^18^^,^^19^ However, biomarkers based on single biological features are likely not enough to identify the patient's response to therapy and survival and allow upfront treatment decisions at diagnosis such as the need to use chemotherapy.

In 2022, we developed and validated the HER2DX genomic test,^20^ a single 27-gene expression and clinical feature-based classifier able to provide 2 independent scores to predict both long-term prognosis and likelihood of pCR in HER2-positive early breast cancer. The assay integrates clinical information (i.e., tumour size and nodal status) with biological information tracking immune response, luminal differentiation, tumour cell proliferation and expression of the HER2 17q12-21 chromosomal amplicon, including the ERBB2 gene.^20^ In the seminal study, the prognostic value of HER2DX was shown in 1341 patients across 5 datasets, and the ability to predict pCR following trastuzumab-based therapy was shown in 558 patients across 4 datasets.^20^ Of note, HER2DX was explored in 91 patients from the PAMELA phase II trial, where patients with early-stage HER2-positive breast cancer received neoadjuvant trastuzumab-lapatinib,^10^ indicating that HER2DX could predict pCR following dual HER2 blockade without chemotherapy. However, it is currently unknown if HER2DX, in the absence of cytotoxic therapy, can predict response to trastuzumab-pertuzumab, the only approved anti-HER2 drug combination in early-stage HER2-positive breast cancer.

Here, we aimed to evaluate the ability of HER2DX to predict efficacy of a de-escalated, chemotherapy-free neoadjuvant regimen in HER2-positive/hormone receptor-positive breast cancer.

## Methods

### Study design and participants

PerELISA is an open label, phase II neoadjuvant study conducted in Italy across 8 institutions^14^ (Supplementary Figure 1). Postmenopausal patients ≥18 years-old were eligible according to the following criteria: previously untreated, histologically confirmed, infiltrating, HER2-positive (immunohistochemistry [IHC] 3+ or in situ hybridization amplification^21^), hormone receptor-positive (oestrogen receptor ≥10% and/or progesterone receptor ≥10%) breast cancer, stage II–IIIA, cardiac ejection fraction within institutional normal range, normal organ and marrow function and availability of tumour tissue suitable for biological/molecular examination before starting treatment. The protocol of the PerELISA study can be found in Supplementary Material.

Patients recruited started letrozole 2.5 mg p.o. daily for 2 weeks followed by a core-biopsy for Ki67 evaluation. Ki67 was evaluated locally by IHC on formalin-fixed paraffin-embedded (FFPE) tissue sections from a diagnostic core biopsy and after 2-weeks of letrozole monotherapy. Patients with tumours showing a relative reduction >20% of Ki67 from baseline were defined as having endocrine sensitive disease (ESD). Patients with tumours showing a relative reduction <20% of Ki67 from baseline, were defined as having endocrine resistant disease (ERD). Patients with ESD received the combination of letrozole, trastuzumab and pertuzumab, according to the following schedule: letrozole 2.5 mg p.o. daily, trastuzumab 8 mg/kg i.v. loading dose in the first cycle, then 6 mg/kg every 3 weeks, pertuzumab 840 mg i.v. loading dose in the first cycle, then 420 mg i.v. every 3 weeks. Trastuzumab and pertuzumab were administered for 5 cycles and letrozole was continued until surgery. Patients with ERD discontinued letrozole and received weekly paclitaxel 80 mg/m^2^ for 13 weeks, combined with pertuzumab and trastuzumab (same dose and schedule as in patients with ESD). Surgery was performed within 3 weeks from last dose of i.v. treatment. No survival follow-up after surgery is available.

### Ethics

The study was performed in accordance with Good Clinical Practice guidelines and the World Medical Association Declaration of Helsinki. All patients provided informed consent. The study was approved by the Istituto Oncologico Veneto's Ethical Committee with internal reference number for the approval **2013/38**.

### Tumour sample procedures

Gene expression assays were performed from pre-treatment baseline FFPE tumour samples at the Translational Genomics and Targeted Therapies in Solid Tumours at IDIBAPS. A minimum of ∼125 ng of total RNA was used to measure the expression of 185 breast cancer-related genes and 5 housekeeping genes (*GAPD, PUM1, ACTB, RPLP0 and PSMC4*) using the nCounter platform (Nanostring Technologies, Seattle, USA).^20^ The gene expression for each sample was independently normalized to the geometric mean of 5 housekeeping genes. The data collected for the study cannot be made publicly available.

### HER2DX 27-gene assay

RNA was obtained from FFPE tumor samples. The HER2DX standardized assay was performed using the nCounter platform (NanoString Technologies, Seattle, WA, USA) as previously described.^20^ The HER2DX assay is based on 4 different gene signatures comprising 27 genes, including the 14-gene immunoglobulin (IGG) module (i.e., *CD27, CD79A, HLA-C, IGJ, IGKC, IGL, IGLV3-25, IL2RG, CXCL8, LAX1, NTN3, PIM2, POU2AF1* and *TNFRSF17*).^22^ The IGG signature has previously shown strong independent prognostic value in a large breast cancer dataset, where patients did not receive adjuvant systemic therapy. The other 3 genes signatures were: a 4-gene tumor cell proliferation signature (*EXO1, ASPM, NEK2* and *KIF23*), a 5-gene luminal differentiation signature (*BCL2, DNAJC12, AGR3, AFF3* and *ESR1*), and the 4-gene HER2 amplicon signature (*ERBB2, GRB7, STARD3* and *TCAP*).^20^ For each signature, the normalized gene expression was calculated for each patient. The HER2DX risk score was calculated based on the IGG, the luminal and the proliferation signatures. The HER2DX pCR likelihood score was calculated based on HER2, IGG, luminal and proliferation signatures. The HER2DX ERBB2 score was calculated based on the ERBB2 mRNA levels. The three HER2DX scores (i.e., risk-score, pCR likelihood score [pCR score] and ERBB2 mRNA score) were reported as continuous variable and according to pre-established cut-offs.^20^

### Outcomes

The primary objective of this study was to test the ability of the three HER2DX scores to predict pCR following letrozole, trastuzumab and pertuzumab in patients with ESD. The secondary objectives were: 1) to test the ability of the three HER2DX scores to predict response to 2-week letrozole monotherapy; 2) to test the ability of the three HER2DX scores to predict response to paclitaxel, trastuzumab and pertuzumab in ERD.

### General statistical procedures

Univariate and multivariable logistic regression analyses were used to investigate the association of each variable (HER2DX risk-score, HER2DX pCR score, HER2DX ERBB2 score or PAM50 HER2-enriched subtype) with pCR. Categorical variables were expressed as number (%) and compared by χ^2^ test or Fisher's exact test. Receiver operating characteristic (ROC) curves were used as a performance measure, DeLong's test was used to compare two ROC curves. The significance level was set to a 2-sided alpha of 0.05. We used R version 4.0.5 for all the statistical analyses.

### Role of the funding source

This study received funding from Reveal Genomics. Funders but also investigators from Padova University, Hospital Clinic and Reveal Genomics participated in the study design, data collection, data analysis, interpretation or writing of report. All authors had full access to all data in the study and had final responsibility for the decision to submit for publication.

## Results

### PerELISA HER2DX study population

**Fig. 1:**
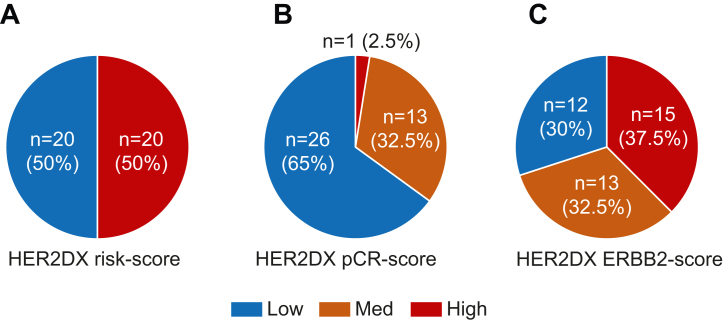
Distribution of the HER2DX scores in patients with ESD recruited in the PerELISA trial. (**a**) HER2DX risk-score; (**b**) HER2DX pCR-score; (**c**) HER2DX ERBB2 mRNA score.

HER2DX was evaluated in 55 patients (86.0%) enrolled in the PerELISA trial (NCT02411344). In terms of baseline clinical-pathological characteristics, median age was 64.0 (49–83) and most patients had clinical stage IIA (n = 37, 67.0%), an adenocarcinoma of no special type (NST) histology (n = 52, 95.0%) and grade 3 disease (n = 41, 77.0%). No statistical differences were observed between the original trial population (n = 61) and this HER2DX study subpopulation (n = 55) (Table 1). Forty (73.0%) patients had ESD and their pCR rate was 22.5% (9/40). In this 40-patient subpopulation with ESD, 50.0% of patients had HER2DX high-risk disease, 65.0% had HER2DX pCR-low disease and 37.5% had HER2DX ERBB2-high disease (Fig. 1). In the 15-patient with ERD, 60.0% of patients had HER2DX high-risk disease, 20.0% had HER2DX pCR-low disease and 60.0% had HER2DX ERBB2-high disease.

**Table 1:** Baseline clinical and tumour characteristics.

	Original trial population			HER2DX trial subpopulation			Statistics
	Overall	Molecular responders	Molecular non-responders	Overall	Molecular responders	Molecular non-responders	
	N = 61	N = 44	N = 17	N = 55	N = 40	N = 15	
**Median age, y (range)**	64 (49–83)	66 (50–83)	60 (49–78)	64 (49–83)	67 (50–83)	62 (49–78)	Two-tailed t-test (all p > 0.05)
**Clinical stage, n (%)**							
IIA	41 (67)	31 (70)	10 (59)	37 (67)	28 (70)	9 (60)	Fisher's exact test
IIB	16 (26)	10 (23)	6 (35)	16 (29)	10 (25)	5 (33)	(All p > 0.05)
IIIA	4 (6)	3 (7)	1 (6)	2 (4)	2 (5)	1 (7)	
**Histology, n (%)**							
Ductal	56 (92)	40 (91)	16 (94)	52 (95)	37 (93)	15 (100)	Fisher's exact test
Lobular/other	5 (8)	4 (9)	1 (6)	3 (5)	3 (8)	0 (0)	(All p > 0.05)
**Histologic grade,**^a^**n (%)**							
G2	14 (24)	13 (30)	1 (6)	12 (23)	12 (31)	0 (0)	Fisher's exact test
G3	45 (76)	30 (70)	15 (94)	41 (77)	27 (69)	14 (100)	(All p > 0.05)

a n = 2 patients do not have histologic grade data.

### HER2DX association with pCR in ESD

**Fig. 2:**
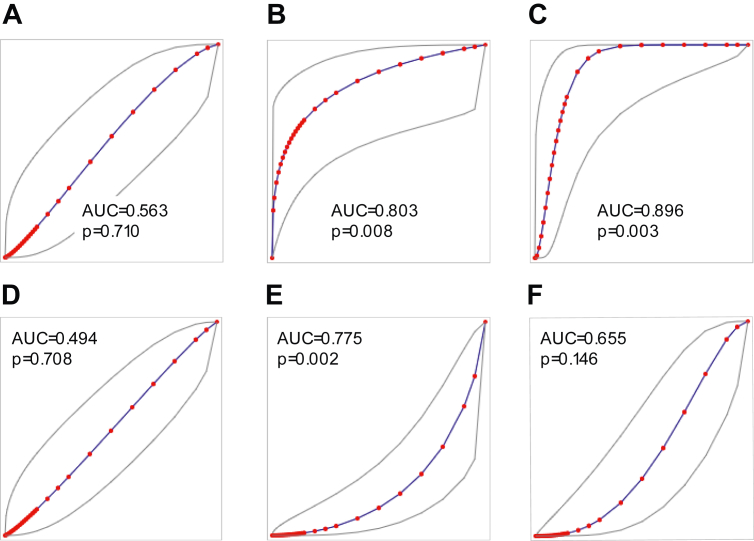
Performance of the HER2DX scores to predict response. (**a**) HER2DX risk-score and pCR in patients with ESD; (**b**) HER2DX pCR-score and pCR in patients with ESD; (**c**) HER2DX ERBB2 mRNA score and pCR in patients with ESD; (**d**) HER2DX risk-score and response to letrozole in all patients; (**e**) HER2DX pCR-score and response to letrozole in all patients; (**f**) HER2DX ERBB2 mRNA score and response to letrozole in all patients. ROC AUC and p-values (univariate logistic regression model) are reported.

HER2DX pCR score and ERBB2 mRNA score as continuous variables were significantly associated with pCR (p = 0.008 and p = 0.003, univariate logistic regression model); area under ROC [AUC] = 0.803 and 0.896), no significant differences were observed between ROC AUC of HER2DX pCR score and ERBB2 mRNA (p = 0.211, DeLong's test) (Fig. 2). The pCR rate in low, medium, and high HER2DX pCR score groups was 7.7% (2/26), 46.2% (6/13) and 100.0% (1/1), respectively (p < 0.004, Fisher's exact test) (Table 2). The pCR rates in low, medium, and high HER2DX ERBB2 mRNA score groups were 0.0% (0/12), 7.7% (1/13) and 53.3% (8/15), respectively (p = 0.001, Fisher's exact test). Consistently, the 7 patients with a pCR and a HER2DX pCR score medium or high, also had a HER2DX ERBB2 high score. Conversely, HER2DX risk-score as a continuous variable was not associated with pCR (p = 0.710, univariate logistic regression model; AUC = 0.563). ROC AUC of HER2DX ERBB2 mRNA was significantly higher than ROC AUC of HER2DX risk-score (p = 0.013, DeLong's test). The pCR rate in low and high HER2DX risk-score groups was 20.0% (4/20) and 25.0% (5/20), respectively (p = 1, Fisher's exact test) (Table 2).

**Table 2:** Association of the three HER2DX scores (risk-score, pCR-score and ERBB2 mRNA) with response to either neoadjuvant letrozole, trastuzumab and pertuzumab or 2-week letrozole in the PerELISA trial.

	Letrozole + Trastuzumab + Pertuzumab (n = 40)			2-week letrozole (n = 55)		
HER2DX risk-score	N	No pCR	pCR	N	No ET response	ET response
High-risk	20	15 (0.0%)	5 (25.0%)	29	9 (31.0%)	20 (69.0%)
Low-risk	20	16 (80.0%)	4 (20.0%)	26	6 (23.1%)	20 (76.9%)
HER2DX pCR score						
pCR-high	1	0 (0.0%)	1 (100.0%)	6	5 (83.3%)	1 (16.7%)
pCR-med	13	7 (53.8%)	6 (46.2%)	20	7 (35.0%)	13 (65.0%)
pCR-low	26	24 (92.3%)	2 (7.7%)	29	3 (10.3%)	26 (89.7%)
HER2DX ERBB2-score						
ERBB2-high	15	7 (46.7%)	8 (53.3%)	24	9 (37.5%)	15 (62.5%)
ERBB2-med	13	12 (92.3%)	1 (7.7%)	17	4 (23.5%)	13 (76.5%)
ERBB2-low	12	12 (100.0%)	0 (0.0%)	14	2 (14.3%)	12 (85.5%)
PAM50 subtype						
HER2-enriched	11	6 (54.5%)	5 (45.5%)	22	11 (50%)	11 (50%)
Others	29	25 (86.2%)	4 (13.8%)	33	4 (12.1%)	29 (87.9%)

ET, endocrine therapy.

We then evaluated the ability of HER2DX pCR score, and HER2DX ERBB2 mRNA score, to predict pCR independently of the PAM50 HER2-enriched subtype. In a previous report, we showed that PAM50 HER2-enriched tumours have a higher pCR rate compared to PAM50 non-HER2-enriched tumours (45.5% vs 13.8%: odds ratio [OR] = 5.21, 95% confidence interval [CI] 1.1–27.6, p = 0.042, multivariable logistic regression model).^8^, ^9^, ^10^, ^11^, ^12^, ^13^, ^14^ In this 40-patient subpopulation with ESD, the PAM50 subtype distribution was 2.5% (1/40) Basal-like, 27.5% (11/40) HER2-enriched, 25% (10/40) Luminal A, 37.5% (15/40) Luminal B and 7.5% (3/40) Normal-like. In a bivariate model that included HER2DX pCR score and the PAM50 HER2-enriched variable, HER2DX pCR score was found significantly associated with pCR (OR = 1.06, 95% CI 1.0–1.12, p = 0.017, bivariate logistic regression model), but not PAM50 HER2-enriched (OR = 1.87, 95% CI 0.3–12.5, p = 0.520, bivariate logistic regression model). In a bivariate model which included HER2DX ERBB2 mRNA score and the PAM50 HER2-enriched variable, HER2DX ERBB2 mRNA score was found significantly associated with pCR (odds ratio [OR] = 1.11, 95% confidence interval [CI] 1.0–1.22, p = 0.006 multivariable logistic regression model), but not PAM50 HER2-enriched (OR = 5.53, 95% CI 0.7–60.03, p = 0.106, multivariable logistic regression model). No other clinical-pathological baseline pre-treatment variable such as tumour stage, nodal stage, oestrogen receptor, progesterone receptor expression, and Ki67 expression was found associated with pCR.

### HER2DX association with 2-week response to letrozole monotherapy

HER2DX pCR score as continuous variables was significantly associated with Ki67 response to 2-weeks of letrozole monotherapy (p = 0.002, univariate logistic regression model; area under ROC [AUC] = 0.775) (Fig. 2). The Ki67-defined response rate in low, medium, and high HER2DX pCR score groups was 89.7% (26/29), 65.0% (13/20) and 16.7% (1/6), respectively (p = 0.001, Fisher's exact test) (Table 2). HER2DX ERBB2 score was not found associated with response to letrozole (p = 0.146, univariate logistic regression model; area under ROC [AUC] = 0.655). The response rate in low, medium, and high HER2DX ERBB2 mRNA score groups was 85.7% (12/14), 76.5% (13/17) and 62.5% (15/24), respectively (p = 0.321, Fisher's exact test). Finally, HER2DX risk-score as a continuous variable was not associated with response to letrozole (p = 0.708, univariate logistic regression model; AUC = 0.494). The response rate in low and high HER2DX risk-score groups was 76.9% (20/26) and 69.0% (20/29), respectively (p = 0.558, Fisher's exact test).

### HER2DX association with pCR in ERD

Fifteen patients (27.0%) had ERD and the pCR rate was 80.0% (12/15). HER2DX risk-score, pCR score and ERBB2 mRNA score as continuous variables were not found associated with pCR following paclitaxel, trastuzumab and pertuzumab (*data not shown*). The pCR rate in low, medium, and high HER2DX pCR score groups was 66.7% (2/3), 85.7% (6/7) and 80.0% (4/5), respectively (p = 1, Fisher's exact test). The pCR rates in low, medium, and high HER2DX ERBB2 mRNA score groups were 50.0% (1/2), 75.0% (3/4) and 88.9% (8/9), respectively (p = 0.499, Fisher's exact test). Finally, the pCR rate in low and high HER2DX risk-score groups was 83.3% (5/6) and 77.8% (7/9), respectively (p = 1, Fisher's exact test).

## Discussion

This study tests the ability of HER2DX to predict response to the combination of trastuzumab and pertuzumab in the absence of cytotoxic therapy in patients with early-stage HER2-positive/hormone receptor-positive breast cancer. Specifically, we found that high HER2DX pCR and ERBB2 mRNA scores were both significantly associated with response to this treatment strategy and were better predictors of response than the previously described PAM50 HER2-enriched subtype. At the same time, we found that a low HER2DX pCR score predicted early Ki67 response to letrozole monotherapy. Finally, we did not observe an association of HER2DX prognostic risk-score with treatment response, confirming prior results^20^^,^^23^^,^^24^ where predictors of long-term outcome in early-stage HER2-positive breast cancer were found to be distinct from predictors of response to neoadjuvant anti-HER2-based treatments.

Several clinical trials have evaluated the use of dual HER2 blockade in early-stage HER2-positive breast cancer in the absence of chemotherapy.^8^, ^9^, ^10^, ^11^, ^12^, ^13^, ^14^ The first trial (i.e., TBCRC-006) was reported by Rimawi and colleagues^8^ in 2013. This study evaluated the combination of neoadjuvant trastuzumab and lapatinib (and endocrine therapy if hormone receptor-positive) for 12 weeks in 66 patients with stage II-III HER2-positive breast cancer and showed a pCR rate of 27.0%. Subsequent neoadjuvant phase II trials such as TBCRC-023^9^ and PAMELA^10^ confirmed these findings. However, the lack of statistical significance in the ALTTO phase III pivotal trial,^25^ which evaluated the combination the addition of lapatinib to trastuzumab compared to trastuzumab, precluded the approval of lapatinib for the treatment of early-stage HER2-positive breast cancer. This unfortunate result limited further exploration of the lapatinib–trastuzumab combination to avoid chemotherapy in early disease. Since then, de-escalation trials of cytotoxic therapy such as PerELISA,^14^ PHERGain^13^ and WSG-ADAPT-HER2-positive/HR-negative^11^^,^^12^ have focused on dual HER2 blockade with trastuzumab and pertuzumab, a combination approved by FDA and EMA for the treatment of early-stage HER2-positive breast cancer. Similar to the lapatinib–trastuzumab combination, the part rates with trastuzumab-pertuzumab range from 20 to 40%.^11^, ^12^, ^13^, ^14^ However, three facts limit the clinical implementation of this treatment strategy. First, the pCR rates when chemotherapy is added to dual HER2 blockade are much higher than without chemotherapy.^12^^,^^15^^,^^26^ Second, the value of pCR in the absence of chemotherapy is still unknown, especially in HER2-positive/hormone receptor-positive disease. Finally, long-term survival outcomes when patients are treated with dual HER2 blockade without chemotherapy are pending. Thus, most patients with stage I-III HER2-positive breast cancer receive chemotherapy today.

The results from the HER2DX pCR score reveal an inverse relationship between endocrine sensitivity and anti-HER2 sensitivity in HER2-positive/hormone receptor-positive breast cancer; namely, the higher the HER2DX pCR score, the more anti-HER2 sensitive the tumour is, and the less endocrine sensitive it is, and vice-versa. The explanation most likely relies on 2 of the 4 gene signatures of the HER2DX assay, namely the luminal signature and the HER2 amplicon signature, which drive the HER2DX pCR score on opposite directions.^20^ Thus, low HER2DX pCR scores are identifying tumours with higher expression of the luminal signature and lower expression of the HER2 amplicon signature, whereas high HER2DX pCR scores identify tumours with lower expression of the luminal signature and higher expression of the HER2 amplicon signature. Previous preclinical studies have described the inverse relationship between the activation of the estrogenic receptor signalling pathway and the HER2 signalling pathway.^27^ Thus, HER2DX pCR score senses this delicate balance between both pathways within HER2-positive/hormone receptor-positive disease.

The level of ERBB2 mRNA was found the best predictor of response to the trastuzumab-pertuzumab combination in the absence of chemotherapy (i.e., AUC = 0.896). Virtually all cases (i.e., 8/9) who achieved a pCR were found to have HER2DX ERBB2 mRNA-high disease, according to the pre-established cut-offs. Thus, in the absence of chemotherapy, the probability of achieving a pCR if the tumour is HER2DX ERBB2 mRNA low or medium, which represents 62.5% of patients with HER2-positive/hormone receptor-positive disease, is only 4.0% (1/25), whereas the probability of achieving a pCR in HER2DX ERBB2 mRNA-high disease is 53.3% (8/15). At the same time, it is important to highlight those 30.0% of patients in PerELISA who had HER2DX ERBB2 mRNA-low tumours. According to HER2DX, ERBB2 mRNA-low tumours should be considered clinically HER2-negative since the distinction between the HER2DX ERBB2 mRNA-low versus ERBB2 mRNA-medium categories is based on an optimal cut-off to identify clinically HER2-positive tumours from HER2-negative tumours, according to the ASCO/CAP HER2 definition.^21^ This is an important finding as the field is moving away from a binary classification of HER2 (i.e., positive versus negative) and new entities are arising such as HER2-low disease,^28^^,^^29^ the latter of which is now being targeted by potent anti-HER2 antibody drug-conjugates. Importantly, we have recently shown that HER2DX ERBB2 score are associated with response to T-DM1 in advanced HER2+ disease regardless of HER2 IHC (2+ vs 3+).^30^ Therefore, robust, and reproducible means of determining the levels of HER2 with a standardized assay with a larger dynamic range of HER2 expression by immunohistochemistry might become necessary soon.

The HER2DX results in PerELISA might also open the door to better select patients for treatment strategies such as trastuzumab-pertuzumab without chemotherapy in early-stage and advanced HER2-positive/hormone receptor-positive breast cancer. In early disease, prospective clinical trials could select patients with HER2DX low-risk disease, HER2DX ERBB2 mRNA-high and HER2DX pCR-high tumours and demonstrate that trastuzumab-pertuzumab treatment without chemotherapy achieves outstanding long-term survival outcomes. In addition, knowing the baseline pre-treatment levels of ERBB2 could help decide whether to continue with dual HER2 blockade (i.e., ERBB2 mRNA-high) or not (i.e., ERBB2 mRNA low or medium) after achieving a pCR after standard anti-HER2-based chemotherapy but further testing is needed. Patients with HER2DX low-risk and ERBB2 mRNA-low or ERBB2 mRNA-medium tumours, the benefit from adjuvant pertuzumab might be very small, if any. In the advanced setting, the PERTAIN phase II clinical trial^31^ showed that first-line trastuzumab-pertuzumab and endocrine therapy without induction of chemotherapy might benefit some patients. However, despite this treatment strategy being recommended by the 5th ESO-ESMO international consensus guidelines for advanced for advanced breast cancer (ABC5) for highly selected patients,^32^ identification of these patients in the clinical setting is not easy without a biomarker. In this context, identification of patients with HER2DX ERBB2 mRNA-high disease could help make the decision to avoid chemotherapy in this context in selected patients. A similar clinical scenario where HER2DX ERBB2 mRNA score might be helpful is for indicating the approved lapatinib–trastuzumab combination^33^ without chemotherapy in late-line.

Our study has some limitations worth noting. First, the PerELISA clinical trial has a small sample size (i.e., n = 55), especially the group of patients with endocrine resistant tumours (i.e., n = 15), which precludes finding significant statistical associations. Second, we used all the samples available from the PerELISA trial for the correlative analysis. However, the lack of formal design through pre-planned analysis prohibits inference of negative results. Second, patients outside from a clinical trial do not typically receive a short course of letrozole monotherapy before starting neoadjuvant therapy. Thus, the PerELISA population is not representative from the general population, yet most patients (i.e., ∼70%) with HER2-positive/hormone receptor-positive breast cancer in PerELISA had endocrine sensitivity tumours. Finally, there is no long-term survival data and the value of pCR in this context is still unclear.

To conclude, HER2DX is a strong predictor of response to endocrine therapy in combination with dual HER2 blockade with trastuzumab-pertuzumab in HER2-positive/hormone receptor-positive early-stage breast cancer. The combination of HER2DX prognostic risk-score, pCR score and ERBB2 mRNA score might help better tailor systemic therapy in this context and identify candidates for avoiding chemotherapy, a therapy associated with short- and long-term toxicities and impact in quality of life. Further studies will delineate the clinical utility of HER2DX in this and other HER2-positive breast cancer settings.

## Contributors

VG and PFC designed the PerELISA trial, and AP designed the HER2DX sub study. AP, PFC, VG, FBM, MVD, GG, LP, FM, MB, CAG, PB, OC and PG contributed to data collection and assembly. AP, LP and FBM analysed the data, and all authors interpreted the data. AP, AV, PV, CMP, JSP, PFC, VG supervised the work. FBM and LP verified the underlying data. All authors wrote and reviewed the report and approved the final version for submission.

## Data sharing statement

The data collected for the study cannot be made publicly available to allow for commercialization of research findings. However, we encourage investigators interested in data access and collaboration to contact the corresponding author (AP). The research-based R code to determine the HER2DX scores are available upon reasonable request to the corresponding author (AP).

## Declaration of interests

Dr. Perou, Dr. Prat, Dr. Vivancos, Dr. Villagrasa, and Dr. Parker are equity stockholders of Reveal Genomics; Dr. Perou, Dr. Prat, Dr. Vivancos, and Dr. Parker are also consultants of Reveal Genomics. Dr. Prat reports grants from Reveal Genomics, during the conduct of the study; other from Reveal Genomics, personal fees from Roche, grants and personal fees from AstraZeneca, grants and personal fees from Daiichi-Sankyo, grants and personal fees from Novartis, personal fees from Foundation Medicine, personal fees from Oncolytics Biotech, outside the submitted work; In addition, Dr. Prat has a patent HER2DX licensed to Reveal Genomics, and a patent WO 2018/103834 licensed to Reveal Genomics. Dr. Paré is an employee of Reveal Genomics and has a patent HER2DX licensed to Reveal Genomics. Dr. Marín-Aguilera is an employee of Reveal Genomics. Dr. Dieci reports personal fees from Astrazeneca, Daiichi Sankyo, Gilead, Eli Lilly, MSD, Exact Sciences, Novartis, Pfizer, Seagen, outside the submitted work; In addition, Dr. Dieci has a patent HER2DX pending to Reveal Genomics. Dr. Griguolo reports personal fees from Eli Lilly, Amgen, Novartis, Pfizer, Daiichi Sankyo, Gileas outside the submitted work. Dr. Guarneri reports personal fees from Eli Lilly, Novartis, MSD, GSK, Gilead, Exact Science, Merck Serno, Sanofi, Pfizer, Amgen, Eisai outside the submitted work; In addition, Dr. Guarneri has a patent HER2DX licensed to Reveal Genomics. Dr. Miglietta reports consulting fees from Gilead outside the submitted work. Dr. Villagrasa reports other from Reveal Genomics, personal fees from Nanostring, outside the submitted work; In addition, Dr. Villagrasa has a patent HER2DX pending. Dr. Conte reports personal fees from Roche, personal fees from Novartis, personal fees from Daiichi Sankyo, personal fees from Astrazeneca, personal fees from Eli-lilly, outside the submitted work; In addition, Dr. Conte has a patent HER2DX pending licensed to Reveal Genomics. Dr. Brasó-Maristany has a patent HER2DX pending licensed to Reveal Genomics. Dr. Vivancos reports personal fees from Bayer, personal fees from Bristol Meyers Squibb, personal fees from Guardant Health, personal fees from Merck, personal fees from Novartis, personal fees from Roche, personal fees from Incyte, outside the submitted work; In addition, Dr. Vivancos has a patent WO2015145388A3 licensed. Dr. Perou are equity stockholders of Bioclassifier LLC and Drs. Perou and Parker have a patent on PAM50 subtype assays; other from Reveal Genomics, outside the submitted work. All the other authors do not report conflicts of interest. No authors have been paid to write this article.
