# Supplementary material for: HER2DX genomic test in HER2-positive/hormone receptor-positive breast cancer treated with neoadjuvant trastuzumab and pertuzumab: A correlative analysis from the PerELISA trial
Source: eBioMedicine. 2022 Oct 29;85:104320. doi: 10.1016/j.ebiom.2022.104320 (PMC9626543; doi:10.1016/j.ebiom.2022.104320)
Supplement: Supplementary Material [file mmc1.pdf]

“**PER-ELISA**: **PER**tuzumab-trastuzumab plus l**E**trozo**L**e **I**n endocrine **S**ensitive breast cancer: a phase II neo**A**djuvant study”

Sponsor:

AS.T.R.O. (ASsociation for Translational Research in Oncology)

Via G. Mameli, 3/1
16122 Genova (GE)

Prof PierFranco Conte

Principal Investigator:

Dr Valentina Guarneri

Address:

University of Padova

Istituto Oncologico Veneto

via Gattamelata 64,

35128 Padova, Italy

Phone +39 049 8215931

Fax: +39 049 8215932

Sponsor signature:...

Principal Investigator signature:...

PROTOCOL SYNOPSIS: PER-ELISA study

Title

"**PER-ELISA**: **PER**tuzumab-trastuzumab plus l**E**trozo**L**e **I**n endocrine **S**ensitive breast cancer: a phase II neo**A**djuvant study"

Indication

Stage II-IIIA operable breast cancer

Study centers

The trial is a multicentric study; 10 Italian Institution will participate to this study.

Objectives

Primary Objective:

- Primary aim is to evaluate the rate of pathologic complete response (pCR), as defined as complete disappearance of invasive tumor in breast and axillary nodes.

Secondary Objectives:

- To estimate the percentage of clinical objective responses (cOR) (complete plus partial) in the breast, as assessed by ultrasonography (US)

- To estimate the percentage of breast conservative surgery

- To evaluate the safety profile

- Correlative biomarker analyses:

    Treatment induced modification in the expression of tumor biomarkers (Ki67, PI3KCA, pTEN, pAKT)

    Relation between baseline biomarker expression and tumor characteristics

    Relation between tumor biomarkers and response

    Relation between tumor gene expression at diagnosis and response

Trial design

This is an open label, muticentric, phase II neoadjuvant study.

After confirmation of eligibility and informed consent signature, the patients will start letrozole 2.5 mg p.o. daily for 2 weeks. After 2 weeks, a biopsy to assess ki67 inhibition will be performed. Letrozole will be continued until the result of Ki67 will be available.

Patients defined as molecular responders (Ki67 reduction ≥ than 20% from baseline at day 14) will start therapy with the combination of letrozole, trastuzumab and pertuzumab, according to the following schedule:

- Pertuzumab 840 mg loading dose iv, followed by 420 mg iv every 3 weeks for 5 cycles

- Trastuzumab 8 mg/kg loading dose iv, followed by 6 mg/kg iv every 3 weeks for four cycles

- Letrozole 2.5 mg daily p.o. for 4 months

Patients defined as molecular non-responders (Ki67 reduction less than 20% from baseline at day 14) will stop letrozole and will receive chemotherapy with weekly paclitaxel in combination with trastuzumab and pertuzumab, at the following doses:

- Pertuzumab 840 mg loading dose iv, followed by 420 mg iv every 3 weeks for 5 cycles

- Trastuzumab 8 mg/kg loading dose iv, followed by 6 mg/kg iv every 3 weeks for four cycles

- Paclitaxel 80 mg/mq for 13 weekly doses

Number of patients

It is planned to enrol approximately 68 patients

Target population

Postmenopausal patients with diagnosis of HER2 positive, hormone receptor positive, stage II-IIIA, previously untreated breast cancer.

Inclusion criteria:

Patients may be included in the study only if they met all the following criteria

- female patients with primary diagnosis of infiltrating breast cancer

- HR positivity (ER ≥ 10% and/or PgR ≥10%) and HER2 positivity (IHC 3+ or FISH/CISH amplification) as assessed by local laboratory

- Stage II-IIIA

- age >18 yrs

- ECOG Performance Status 0-1

- Postmenopausal status, defined by at least one of the following:

  60 years of age;

  < 60 years of age and amenorrheic for >/=12 months prior to day 1

  < 60 years of age, without a uterus, and luteinizing hormone (LH) and follicle stimulating hormone (FSH) values within postmenopausal range

  Prior bilateral oophorectomy

  Prior radiation castration with amenorrhea for at least 6 months

- Cardiac ejection fraction within the institutional range of normal (as measured by echocardiogram or MUGA scan).

- Normal organ and marrow function as defined below:

  (leukocytes >=3000/mcL;

  absolute neutrophil count >=1,500/mcL;

platelets >=100,000/mcL;

total bilirubin within 1.25 x normal institutional limits (with the exception of Gilbert's syndrome);

AST (SGOT)/ALT(SGPT) within 1.25 x institutional upper limit of normal

creatinine within normal institutional limits

- Availability of tumor tissue suitable for biological and molecular examination before starting primary treatment

- Ability to understand and the willingness to sign a written informed consent document.

Exclusion criteria:

Patients will be excluded from the study for any of the following reasons

- Stage IIIB, IIIC, and inflammatory breast cancer

- Stage IV breast cancer

- Prior treatment with chemotherapy, endocrine therapy or radiotherapy. Prior treatment with HER2 targeting therapies

- LVEF below the ULN

- Uncontrolled hypertension (systolic >150 mm Hg and/or diastolic >100 mm Hg) or clinically significant (i.e. active) cardiovascular disease: cerebrovascular accident (CVA)/stroke or myocardial infarction within 6 months prior to first study medication, unstable angina, congestive heart failure (CHF) of New York Heart Association (NYHA) grade II or higher, or serious cardiac arrhythmia requiring medication.

- Received any investigational treatment within 4 weeks of study start.

- Subjects with known infection with HIV, HBV, HCV

- Known hypersensitivity to any of the study drugs or excipients.

- Dyspnea at rest or other disease requiring continuous oxygen therapy.

- Psychiatric illness/social situations that would limit compliance with study requirements

- Subjects assessed by the investigator to be unable or unwilling to comply with the requirements of the protocol

Length of the study

It is planned to enrol approximately 68 patients over about 24 month. The study will end after the last patients surgery.

Study period

Estimated date of first subject enrolled: September 2013

Estimated date of last subject completed: March 2016

Duration of treatment

Trastuzumab+Pertuzumab (plus weekly paclitaxel in molecular non responders) will be administered for five 21-day cycles prior to surgery, that will be performed within 3 weeks from the last iv therapy infusion.

Study treatment will be stopped in case of disease progression, unacceptable toxicity, withdrawal of consent, or death. Patients will be followed until surgery.

Investigational medical product(s)

Pertuzumab is considered to be the investigational medicinal product in this study.

Non-investigational medical product(s)

Trastuzumab, letrozole and paclitaxel are considered to be non-investigational medicinal products in this study.

Dose/route/regimens

After confirmation of eligibility and informed consent signature, the patients will start letrozole 2.5 mg p.o. daily for 2 weeks, then a biopsy to assess Ki67 will be performed.

<u>Patients defined as molecular responders will start therapy with the combination of letrozole, trastuzumab and pertuzumab, according to the following schedule:</u>

- Pertuzumab 840 mg loading dose iv on day one, followed by 420 mg iv every 3 weeks for 5 cycles

- Trastuzumab 8 mg/kg loading dose iv on day 2, followed by 6 mg/kg iv on day 2 of each subsequent 3 weekly cycle

- Letrozole 2.5 mg daily p.o. for five 21-day cycles (to be continued until surgery).

If no reactions on cycle 1, pertuzumab and trastuzumab will be administered on the same day (day 1) for the following 4 cycles.

Patients defined as molecular non-responders will stop letrozole and will receive chemotherapy according to the following schedule:

- Pertuzumab 840 mg loading dose iv on day one, followed by 420 mg iv every 3 weeks for 5 cycles

- Trastuzumab 8 mg/kg loading dose iv on day 2, followed by 6 mg/kg iv on day 1 of each subsequent 3 weekly cycle

- Paclitaxel 80 mg/sqm iv administered on a weekly basis starting from day 2 for 13 weeks

Standard premedication will be administered prior to each iv infusion.

If no reactions on cycle 1, pertuzumab, trastuzumab and paclitaxel will be administered on the same day (day 1) for the following 4 cycles

Endpoint evaluation

<u>Pathologic response:</u>

A pathologic complete response (pCR) is defined the complete absence of infiltrating tumor cells in the breast and in lymph nodes. Residual in situ disease (DCIS) will be included in the pCR category.

Clinical response:

The Clinical Response will be defined comparing the tumor size (largest tumor diameter) before and after treatment, as assessed by ultrasound examination, according to RECIST criteria.

Conservative surgery:

The rate of conservative surgery will be calculated as the percent of the conservative procedures performed after primary therapy. The proportion of patients achieving breast conserving surgery (BCS) will be tabulated. The conversion from mastectomy to BCS will be calculated as the percent difference between the number of conservative surgical procedures feasible at study entry and the number of the conservative procedures performed after primary therapy

Safety

To assess the safety profile, nature, incidence and severity of adverse events (AEs) and serious adverse events (SAEs) will be collected. Incidence of and reasons for study drug dose interruption or reduction and discontinuation will be collected. Toxicities will be graded using NCI Common Terminology Criteria for Adverse Events (CTCAE) version 4.0.

All patients who received at least one dose of treatment will be included in the safety evaluation.

Summary of Study Procedures and Assessments

Within 4 weeks prior to first dose

• Subject to sign the informed consent form (ICF)

• Ascertain availability of tumor tissue for biomarker analyses

• Radiologic tumor assessment (mammogram and ultrasound)

• Routine work up staging (liver US, chest XR, bone scan and CT scan when clinically indicated)

• Record any non-serious and serious AEs and assign appropriate toxicity grade

Within 2 weeks prior to first dose

• Physical examination including tumor measurement (by calliper)

• Surgeon’s statement of the surgical procedure needed without primary CT

• Medical history, concomitant medications, ECOG Performance Status

• Electrocardiogram (12-lead ECG) ; Echocardiogram or MUGA scan (note that baseline and on treatment scans should be performed using the same modality and preferably at the same institution)

• Vital signs (blood pressure and pulse rate, body temperature, height, and body weight)

• Hematology and blood chemistry

• Record any non-serious and serious AEs and assign appropriate toxicity grade

Every 3 weeks

The following assessments are to be performed every 3 weeks

• Hematology and blood chemistry

• Physical examination, including tumor measurement (by calliper)

• ECOG Performance Status; Vital signs (blood pressure and pulse rate, body temperature, and body weight)

• Record any non-serious and serious AEs and assign appropriate toxicity grade

• Record all concomitant medication(s) added and/or changed

• A 3-week supply of letrozole will be dispensed to the patient

Assessments at Study Conclusion or Withdrawal from Therapy

The following assessments will be performed at the end of the treatment period and if possible, when a subject is prematurely withdrawn from study for any reason:

• Physical examination, including tumor measurement (by calliper)

• Tumor measurement by mammogram and ultrasonography

• Vital signs (blood pressure and pulse rate, body temperature, and body weight)

• ECOG Performance Status

• Electrocardiogram (12-lead ECG); Echocardiogram or MUGA scan

• Record any AE(s) and serious adverse event(s) (SAEs) and assign appropriate toxicity grade

• Record all concomitant medications(s) added and/or changed

• Hematology and blood chemistry

• Chest XR and liver US

Statistical sections

Sample size has been estimated by using the optimal Simon's two-stage design. Assuming in Ki67 responders a pCR rate of < 10% with endocrine therapy alone, and of around 15% with dual antiHer2 therapy (pertuzumab + trastuzumab, without endocrine therapy), the combination of endocrine therapy + pertuzumab + trastuzumab will be considered worthwhile in case of a pCR rate= 25%.

Suppose that we want to test

H0 : 0.10

H1: 0.25

- With α = 0.05 and β = 0.10 (power = 0.90), in stage I, n 18 patients are accrued. If </= 2 pCR are observed, then the trial is stopped for futility.

- Otherwise additional n =25 patients are accrued, for a total of 43 patients. If at least 8 pCRs are observed, further investigation (i.e, Phase III trial) is warranted

Assuming that approximately 70% of the patients will have molecular response, a total of 68 patients will be included to allow 48 patients in the hormonal therapy arm, with 10% drop off.

The patients not having a molecular response, thus receiving paclitaxel plus trastuzumab-pertuzumab, will be followed and the pCR rate evaluated as internal control.

Biomarkers section

The tumor samples (block or tissue slices) will be centrally collected in a Tumor tissue bank. The following biomarkers will be centrally evaluated: pAKT, pTEN, ki67. Treatment allocation after 2 weeks of will be performed on the basis of local laboratory Ki 67 assessment, and will be centrally reviewed.

Gene mutation analyses will be carried-out to study the occurrence of mutations in the PIK3CA gene. Fresh tumor tissue from core-biopsy must be snap frozen, to perform a microarray analysis of the gene expression profile before treatment and to evaluate its correlation with response.

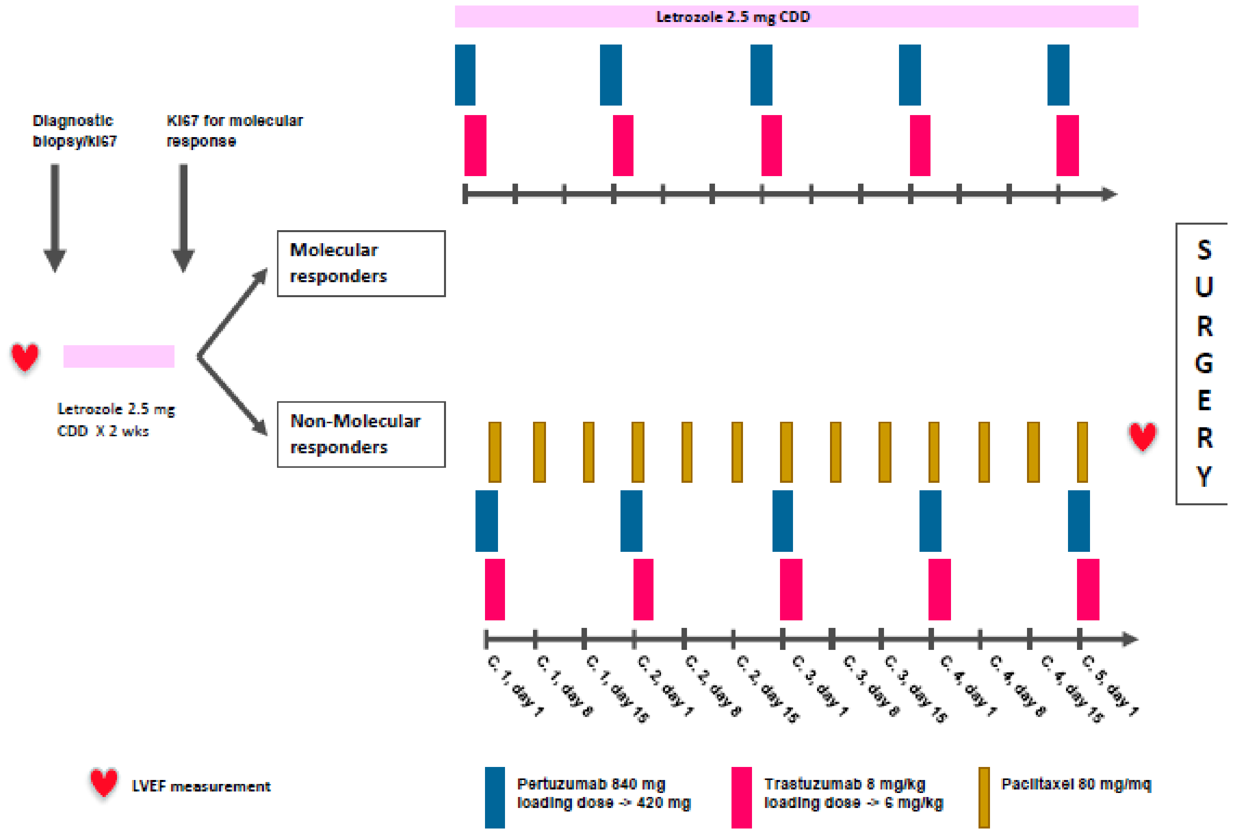

	Screening		Treatment period (Every 3 wks)	pre-Surgery 2 wks after end of treatment	WD
**Day/Day of cycle/Week**	**-28 to 0**	**-14 to 0**			
Informed consent	X				
Medical history		X			
Demography	X				
Inclusion/exclusion criteria	X	X			
Liver US, Chest XR	X			X	
Bone Scan/CT scan (when clinically indicated or in case of positive axillary nodes)	X				
Physical examination (vital signs, performance status and weight)		X	X	X	X
Concurrent medication		X	X	X	X
Histological diagnosis (biopsy)	X				
Biopsy for biomarkers	X				
Tumor assessment (clinical)		X	X	X	
Tumor assessment (ultrasonography)	X			X	
Tumor assessment (mammography)	X			X	
Pathological tumour assessment				X	
Haematology /Blood chemistry		X	X	X	X
Electrocardiogram (ECG)/ECHO or MUGA		X	to be repeated if clinically indicated	X	X
Adverse events	X	X	X	X	X
Dispense letrozole			X		

TABLE OF CONTENTS PAGE

PROTOCOL SYNOPSIS: PER-ELISA STUDY 3
LIST OF ABBREVIATIONS AND DEFINITION OF TERMS 14
INVESTIGATORS AND FACILITIES 16
STUDY LOCATIONS 16
STUDY MANAGEMENT 16
Principal Investigator 16
Coordinating Centre 16
Statistician 16
Sponsor 16
Funding and resources 17
1. INTRODUCTION 18
1.1 BREAST CANCER 18
1.2 STUDY TREATMENTS 18
1.2.1 TRASTUZUMAB IV (RHUMAB HER2,HERCEPTIN®) 18
1.2.3 PERTUZUMAB (RHUMAB 2C4) 20
1.3 BACKGROUND AND RATIONALE FOR THE STUDY 21
1.3.1 Rationale for preoperative treatment 21
1.3.2 Rationale for preoperative endocrine therapy in HR+ breast cancer 21
1.3.3 Preoperative therapy in HER2-positive disease: rationale for dual anti-HER2 inhibition 22
1.3.4 Rationale for combining hormone therapy and antiHER2 agents for HR+/HER2+ 23
1.3.5 Rationale for the study 24
2. STUDY OBJECTIVES 26
2.1 PRIMARY OBJECTIVE 26
2.2 SECONDARY OBJECTIVES 26
3. STUDY PLAN AND PROCEDURES 26
3.1 OVERALL STUDY DESIGN AND FLOW CHART 26
3.2 STUDY PROCEDURES AND ASSESSMENTS 29
3.3 LABORATORY ASSESSMENT 31
3.4 BIOMARKERS EVALUATION 31

4. STUDY POPULATION 32
4.1 OVERVIEW 32
4.2 INCLUSION CRITERIA....... 32
4.3 EXCLUSION CRITERIA 33
4.4 OTHER ELIGIBILITY CRITERIA CONSIDERATIONS 33
4.5 DISCONTINUATION OF SUBJECTS FROM TREATMENT AND ASSESSMENT 34
4.5.1 Criteria for discontinuation 34
4.5.2 Procedures for discontinuation 34
5 TREATMENTS 35
5.1 NON INVESTIGATIONAL PRODUCTS 35
5.1.1 Letrozole 35
5.1.2 Trastuzumab 35
5.1.3 Paclitaxel 35
5.2 INVESTIGATIONAL PRODUCTS 35
5.2.1 Pertuzumab 35
5.3 DOSES AND TREATMENT REGIMENS 35
5.4 DOSE MODIFICATIONS, INTERRUPTIONS OR DELAYS 37
5.4.1 Letrozole 37
5.4.2 Paclitaxel 37
5.4.3 Trastuzumab and Pertuzumab 38
5.5 STORAGE AND ACCOUNTABILITY 42
5.6 PRE-STUDY, CONCOMITANT, AND POST-STUDY TREATMENT(S) 42
5.7 TREATMENT COMPLIANCE 43
6. SAFETY ASSESSMENT 44
6.1. GENERAL SAFETY ASSESSMENT 44
6.2 DRUG – ADVERSE EVENT RELATIONSHIP 45
6.3 CARDIAC SAFETY ASSESSMENT 46
6.4 SAFETY PARAMETERS AND DEFINITION 46
6.4.1 Adverse Events (AE) 47
6.4.2 Laboratory Test Abnormalities 47
6.4.3 Serious Adverse Events (SAEs) 48

6.5 HANDLING OF SAFETY PARAMETERS 49
6.5.1 Reporting of Adverse Events 49
6.5.2 Reporting of Serious Adverse Events (Immediately Reportable) 49
6.5.3 Reporting of Non-Serious Adverse Events of special interest 49
6.5.4 Progression of Underlying Malignancy 50
6.5.5 Follow-up of AEs 51
7. STATISTICAL SECTION, MEASUREMENTS OF STUDY VARIABLES , DATA COLLECTION AND MANAGEMENT 52
7.1 SAMPLE SIZE 52
7.2 MEASUREMENTS OF STUDY VARIABLES 52
7.2.1 Clinical Response 52
7.2.2 Pathologic Response 53
7.2.3 Rate of Conservative Surgery 53
7.2.4 Safety measurements and variables 53
7.2.5 Biomarkers Evaluation 53
7.3 DATA COLLECTION AND MANAGEMENT, QUALITY ASSURANCE 54
7.4 CHANGES TO THE PROTOCOL 54
8. ETHICS 55
8.1 ETHICS REVIEW 55
8.2 ETHICAL CONDUCT OF THE STUDY 55
8.3 WRITTEN INFORMED CONSENT 55
8.4 SUBJECT DATA PROTECTION 55
9. STUDY CONTACT FOR PROTOCOL-RELATED INQUIRIES 56
10. EMERGENCY PROCEDURES 56
10.1 PROCEDURES IN CASE OF MEDICAL EMERGENCY 56
10.2 PROCEDURES IN CASE OF OVERDOSE 56
11. USE OF DATA AND PUBLICATIONS POLICY 56
12. RISKS AND BENEFITS OF PARTICIPATING PATIENTS 57
13. REFERENCES 58

LIST OF ABBREVIATIONS AND DEFINITION OF TERMS

The following abbreviations and special terms are used in this study protocol.

Abbreviation or special term	Explanation
AE	Adverse event
ALP	Alkaline phosphatase
ALT	Alanine aminotransferase
ANC	Absolute neutrophil count
Assessment	An observation made on a variable involving a subjective judgement (assessment)
AST	Aspartate aminotransferase
BUN	Blood urea nitrogen
CI	Confidence interval
CHF	Congestive Heart Failure
CRF	Case report form
CT	Computerised tomography
CTC	Common toxicity criteria
DQS	Data query sheet
ECG	Electrocardiogram
EDTA	Ethylene diamine tetra-acetic acid
EGFR	Epidermal growth factor receptor
ER	Oestrogen receptor
GCP	Good Clinical Practice
GI	Gastrointestinal
HDPE	High density polyethylene
HIV	Human immunodeficiency virus
IB	Investigator's Brochure
ICH	International Conference on Harmonisation
IEC	Independent Ethics Committee
IHC	Immunohistochemistry
ILD	Interstitial lung disease
INR	International Normalised Ratio
IRB	Institutional Review Board
ITT	Intention-to-treat
iv	Intravenous

Abbreviation or special term	Explanation
L/l	Litre
LLT	Low level term
mg	Milligram
ml	Millilitre
MUGA	Multigated acquisition
NCI	National Cancer Institute
NYHA	New York Heart Association
Outcome variable	A variable (usually a derived variable) specifically defined to be used in the analysis of a study objective.
Parameter	A quantity (usually unknown) that characterises the distribution of a variable in a population of subjects.
pCR	Pathological complete response
PgR	Progesterone receptor
Principal investigator	A person responsible for the conduct of a clinical study at an investigational study site. Every investigational study site has a principal investigator.
PS	Performance status
PT	Prothrombin time
RECIST	Response Evaluation Criteria In Solid Tumours
SAE	Serious adverse event
SC	subcutaneous
ULRR	Upper limit of reference range
ULN	Upper limit of Normal
WHO	World Health Organisation

INVESTIGATORS AND FACILITIES

Study Locations

Oncology Unit of the Istituto Oncologico Veneto. Via Gattamelata, 64. Padua. Italy.
Head: dr PierFranco Conte
Tel: 049 8215931
Fax: 049 8215932
This Unit is equipped with all the facilities to deliver the chemotherapy treatment.

A list of participating clinical sites where the trial will be conducted is provided in appendix 1.

Study Management

Principal Investigator

Dr Valentina Guarneri
Oncology Unit – IOV-IRCCS - University of Padova
Tel: 049 8215291, Email: valentina.guarneri@unipd.it

Coordinating Centre

Clinical Trial Unit of the Istituto Oncologico Veneto
Head: dr Gian Luca De Salvo
Tel: 049 8215704
Fax: 049 8215706
This Unit serves as data centre for the clinical trial and provides all the aspects of data management and analysis.

Statistician

Dr Paola Del Bianco, Clinical Trials and Biostatistics Unit – IOV-IRCCS
Tel: 049 8215772, Email: paola.delbianco@ioveneto.it

Sponsor

AS.T.R.O. (ASsociation for Translational Research in Oncology)
Via G. Mameli, 3/1
16122 Genova (GE)

Prof PierFranco Conte

Funding and resources

The Sponsor will finance the study utilizing appropriate research funds and will be the only proprietary of the data collected throughout the study. Roche SpA, proprietary of the investigational drug, will be responsible for providing the study drug. Moreover, Roche SpA will provide some economical support and/or other resources necessary to carry out the study.

1. INTRODUCTION

1.1 Breast Cancer

Breast cancer is the most commonly diagnosed cancer and the second most common cause of cancer mortality in women. Recent epidemiological data have shown a significant decline in breast cancer mortality over the past 15 years, as a result of screening programs, better education, and the introduction of more effective adjuvant treatments [Jemal et al. 2011]. Unfortunately, about 25%–40% of patients eventually develop metastatic disease that is still largely incurable. [Howlader et al. 2008] Today, an important determinant of treatment effects is the molecular characterization of breast cancer. Breast cancer consists of at least three different diseases: hormone-sensitive breast cancer (which represent almost 65% of breast cancers), the human epidermal growth factor receptor (HER)2-positive subtype (15-20%), and triple-negative disease. Each molecular subtype has distinct biological features, distinct clinical courses and requires distinct therapeutic approaches. Although chemotherapy is the only available option so far for the triple-negative subtype, which is characterized by the absence of hormone receptors and HER-2 negativity, endocrine manipulation is the cornerstone of therapy for hormone receptor–positive tumors, and the incorporation of anti–HER-2 agents has become the mainstay of treatment for the HER2-positive subgroup. Indeed, HER2 overexpression has been independently associated with poorer disease-free survival and overall survival (OS) compared with tumors that do not overexpress HER2 [Pauletti et al. 2000]. Since the introduction of anti-HER2 agents, first of all the monoclonal antibody trastuzumab, the prognosis of this subset of breast cancer significantly improved. [Dawood et al. 2010]

1.2 Study Treatments

1.2.1 TRASTUZUMAB IV (RHUMAB HER2,HERCEPTIN®)

Trastuzumab (Herceptin) is a humanized monoclonal antibody directed against the extracellular domain of HER2. The linkage of H with the receptor blocks its activation, which in turn induces an arrest in the downstream intracellular transduction pathway and prevents the transcription

of related genes. Trastuzumab is approved for the treatment of HER2-positive breast cancer in both the early and the advanced settings.

In HER2 + advanced disease, the combination of Trastuzumab and chemotherapy resulted in 25% survival increase (from 20 to 25 months median survival), as compared to chemotherapy alone. [Slamon et al. 2001, Marty et al. 2005] The benefit of incorporating trastuzumab in the treatment of HER2+ breast cancer has been translated also in the early setting. At MDACC, patients with HER2 positive operable breast cancer received upfront chemotherapy (4 courses of paclitaxel followed by 4 courses of FEC - fluorouracil, epidoxorubicin and cyclophosphamide) with or without concomitant Trastuzumab. The rate of pathological complete response (pCR) was 26.3% without and 66.7% with H respectively. [Buzdar et al. 2005] Recently, a Cochrane meta-analysis has evaluated the effect of the addition of Trastuzumab to adjuvant chemotherapy in HER2+ breast cancer patients. Eight studies involving 11,991 patients were included. The combined HRs for overall survival (OS) and disease-free survival (DFS) significantly favoured the trastuzumab-containing regimens (HR 0.66; 95% confidence interval (CI) 0.57 to 0.77, $P < 0.00001$; and HR 0.60; 95% CI 0.50 to 0.71, $P < 0.00001$, respectively). [Moja et al., 2012]

Trastuzumab is well tolerated both as a single agent and in combination with standard chemotherapy. The most common (occurring in ≥1 out of 10 treated patients) adverse reactions are infusion-associated symptoms such as fever and chills, usually following the first infusion of trastuzumab IV. These symptoms are usually mild to moderate in severity and occur infrequently with subsequent trastuzumab IV infusions in up to 40% of patients.

The most significant adverse event (AE) observed in patients who received trastuzumab was cardiac dysfunction, reflected by asymptomatic decreases in left ventricular ejection fraction (LVEF) and, less frequently, by clinically symptomatic congestive heart failure (CHF). In the previously mentioned Cochrane meta-analysis of trastuzumab adjuvant trials, Trastuzumab significantly increased the risk of congestive heart failure (CHF: RR 5.11; 90% CI 3.00 to 8.72, $P < 0.00001$) and left ventricular ejection fraction decline (LVEF: RR 1.83; 90% CI 1.36 to 2.47, $P = 0.0008$). [Moja et al. 2012] Risk factors for cardiac failure in the setting of trastuzumab treatment include coadministration with anthracycline-based chemotherapy, increasing age,

declining LVEF during treatment to below the lower limit of normal, and the use of anti-hypertensive medications [Tan-Chiu et al. 2005].

In addition, severe hypersensitivity reactions have been infrequently reported in patients treated with trastuzumab IV (the exact incidence of these events is unknown). Signs and symptoms include anaphylaxis, urticaria, bronchospasm, angioedema, and/or hypotension. In some cases, the reactions have been fatal. Symptom onset generally occurred during an infusion, but onset after the completion of an infusion has also been reported. Reactions were most commonly reported in association with the initial infusion

1.2.3 PERTUZUMAB (RHUMAB 2C4)

Pertuzumab is a first-in-class recombinant, humanized monoclonal antibody that binds to a different extracellular HER2 site as trastuzumab does. Pertuzumab binds to domain II of the HER2 receptor [Franklin et al. 2004], thus inhibiting HER2 heterodimerization with HER1, HER3, and HER4. Due to the different binding sites of pertuzumab and trastuzumab, ligand-activated downstream signalling is blocked by pertuzumab, but not by trastuzumab. Due to their complementary modes of action, there is a potential role for the combination of pertuzumab and trastuzumab in HER2-overexpressing diseases. Phase II trial data reported only a modest activity for pertuzumab monotherapy in heavily pre-treated HER2-positive MBC patients and in HER2-negative patients. [Cortés et al. 2009, Gianni et al. 2010] However, pertuzumab combined with trastuzumab has shown an overall RR of 24% in 66 HER2-positive MBC patients who progressed under trastuzumab. [Baselga et al. 2010).

In patients with a range of tumor types who received pertuzumab with docetaxel every 3 weeks until progressive disease or unacceptable toxicity (Study BO17021), the maximum tolerated dose of docetaxel when given with pertuzumab was determined to be 75 mg/m2. Dose-limiting toxicities were observed, including one case of Grade 4 febrile neutropenia and one case of Grade 3 fatigue. [Attard et al. 2007]

1.3 Background and Rationale for the study

1.3.1 Rationale for preoperative treatment

Preoperative chemotherapy (PCT) is now considered the standard treatment for locally advanced and inflammatory breast carcinoma, a reasonable approach for operable breast cancer with unfavourable breast-tumor ratio and an acceptable alternative for all early breast cancer patients candidate to adjuvant chemotherapy. The up-front utilization of primary systemic therapy can downstage the primary tumor in the majority of patients, thus allowing for breast conservative surgery, and the achievement of a pathologic complete response is a powerful surrogate of long-term disease-free survival. Furthermore, PCT represents an interesting research tool which allows to design tailored treatments based on the observed response, and to identify tumor biomarkers with prognostic and/or predictive value.
[Rastogi et al. 2008]

1.3.2 Rationale for preoperative endocrine therapy in HR+ breast cancer

The majority of preoperative chemotherapy trials have shown that the probability of obtaining a complete response to chemotherapy is significantly lower in case of hormone sensitive tumors, thus suggesting a major role for endocrine manipulation for these patients. As compared to tamoxifen, which has been the standard endocrine agent for several years, the aromatase inhibitors (AIs) have shown superiority in both advanced and early disease [The ATAC Trialists' Group. 2002, Thurlimann et al. 2005, Coombes et al. 2004, Eiermann et al. 2001, Ellis et al. 2001]. Semiglazov et al. reported a direct comparison between neoadjuvant chemotherapy and neoadjuvant endocrine therapy with aromatase inhibitors, in 239 postmenopausal ER positive and/or PgR positive breast cancer patients, which were randomized to either doxorubicin and paclitaxel every three weeks for four cycles or to anastrozole or exemestane. The results showed no significant differences among the groups in terms of clinical overall response, pCR rates and disease progression. Breast conservative surgery rates were slightly higher in the AI groups compared to chemotherapy. [Semiglazov et al. 2007] In view of these data, preoperative hormonal therapy with letrozole is a reasonable approach in case of hormone sensitive tumors,

allowing to consider the indication for cytotoxic agents in case of non satisfactory response, and to spare unnecessary toxicity from chemotherapy in case of responsive tumors.

Moreover, the evaluation of tumor biomarkers (i.e. Ki 67) permits to early identify those patients who will more likely benefit from treatment. For instance, the IMPACT trial randomized 330 postmenopausal patients to receive preoperative anastrozole or tamoxifen or the association of both drugs: no differences were observed in terms of overall responses and breast conserving surgery, which are classical parameters for chemotherapy efficacy. However, a greater inhibition of tumor proliferation was observed in the anastrozole arm as compared to tamoxifen or to the combination, anticipating on few hundreds of patients the results of the large adjuvant ATAC trial [Dowsett et al. 2005]. More interestingly, Ki-67 levels after two weeks of therapy was an independent predictive factor for relapse free survival. [Smith et al. 2005, Dowsett et al. 2007]

Another illustration of the role of Ki67 in this context derives from the phase II study that evaluated the association of preoperative letrozole and everolimus. In this trial, everolimus (RAD001) has demonstrated to significantly increase letrozole efficacy in neoadjuvant therapy of 270 patients randomly assigned to four months of letrozole and either everolimus (10 mg/day) or placebo: response rate by clinical palpation in the everolimus arm was higher than that with letrozole alone (68.1% versus 59.1%). A reduction in Ki-67 expression after 15 days of treatment occurred in 57% of patients in the everolimus arm and in 30% of patients in the placebo arm ($p<0.01$). [Baselga et al. 2009]

1.3.3 Preoperative therapy in HER2-positive disease: rationale for dual anti-HER2 inhibition

Nowadays, the treatment of early HER2 positive breast cancer requires the administration of trastuzumab in combination with chemotherapy; however, recent data support the feasibility and efficacy of dual HER2 blockade with the concomitant administration of two molecularly targeted agents without chemotherapy. One of the first evidences in this context came from the metastatic setting. In a randomized study, patients who experienced disease progression on prior trastuzumab received either trastuzumab plus lapatinib or single-agent lapatinib. Patients in the combination arm showed a more prolonged PFS (HR 0.73; 95%CI 0.57-0.93, p=0.008). [Blackwell et al. 2012] The recent first-line randomized phase III trial CLEOPATRA, including 808

HER2-positive MBC patients, reported a significant advantage in terms of PFS for the combination of trastuzumab, pertuzumab and docetaxel as compared to the combination of trastuzumab and docetaxel (PFS 18.5 vs 12.4 months, HR 0.62; 95% CI, 0.51 to 0.75; P<0.001). A strong trend in prolongation of OS was also shown, based on an exploratory interim analysis. No increase in cardiotoxicity with the addition of pertuzumab was observed. [Baselga et al. 2012]

A growing body of knowledge on dual HER2 blockade is deriving from the neoadjuvant setting. In the phase III NeoALLTO study, the combination of trastuzumab, lapatinib and chemotherapy resulted in the highest pCR rate (51%) as compared to the addition of either trastuzumab (29.5%, p<0.00001) or lapatinib (24.7%) to chemotherapy. [Baselga et al. 2012] The phase II randomized CHERLOB study evaluated the activity of neoadjuvant lapatinib, trastuzumab or both in combination with an anthracycline-taxane sequential chemotherapy. A total of 121 patients were enrolled. Consistently with the NeoALLTO data, the pCR rate was increased when the dual anti-HER2 blockade was added to chemotherapy (46.7%, 25% and 26.3% for the combination, trastuzumab and lapatinib arms, respectively). [Guarneri et al. 2012] In the phase II randomized NeoSphere trial, the addition of pertuzumab to docetaxel-trastuzumab based primary chemotherapy increased the pCR from 29% to 46%. Interestingly, those patients who received trastuzumab and pertuzumab only, without chemotherapy, experienced a 16.8% pCR rate. [Gianni et al. 2012] Therefore, although the best results are still obtained when chemotherapy is added, data from the non-chemotherapy arm of the NeoSphere trial suggest that a proportion of Her2-positive breast cancer patients may be cured with dual HER2 inhibition alone and they can be spared unnecessary toxicity of cytotoxic agents.

1.3.4 Rationale for combining hormone therapy and antiHER2 agents for HR+/HER2+

Approximately half of hormone receptor positive breast cancer over-expresses HER2 receptor. Important bidirectional crosstalk occurs between the two pathways. Adjuvant trials evaluating the addition of trastuzumab to chemotherapy demonstrate a longer disease free survival for patients with HER2-positive, ER-positive cancers, compared to those with HER2-positive, ER-negative cancers, despite a similar benefit of adding trastuzumab to chemotherapy in both groups. [Gianni et al. 2011, Perez et al. 2011] Clinical trials [Baselga et al. 2012, Guarneri et al. 2012, Gianni et al. 2012, Chang et al. 2011, Holmes et al. 2011, Untch et al. 2012] which

evaluated combinations of HER2-targeted agents with or without chemotherapy as pre-operative therapy for HER2- positive breast cancers demonstrate a lower PCR in patients with tumors concomitantly expressing ER, compared to ER-negative cancers. Despite the fact that PCR rates are lower in ER-positive, compared with ER-negative, HER2-positive breast cancers treated with HER2-directed therapies, PCR rates in ER-positive cancers range from 16 % to as high as 71 %. In the NeoSphere trial, the pCR rate ranges from 29% to 63% in HR-/Her2+ and from 6 to 26% in the HR+/HER2+ subgroups. In particular, the combination of trastuzumab, pertuzumab and docetaxel resulted in a 26% pCR rate in HR+/Her2+ versus 63% in HR-/Her2+ patients. [Gianni et al. 2012] These findings may indicate that ER-positive, HER2-positive cancers are heterogeneous in nature and suggest the existence of a subset of ER+/HER2-positive cancers that have a natural history more aligned with ER-positive, HER2- negative breast cancers. On these premises, targeting both hormone receptors and EGFR signalling pathways represents an attractive therapeutic option for this particular subset of breast cancer, with the aim of optimizing endocrine therapy and possibly overcoming the occurrence of resistance.

This approach has become a valid option in the metastatic setting. The randomized phase II TAnDEM trial included 207 patients with known HER2-positive/ER-positive MBC and reported a doubling of PFS with the addition of trastuzumab over anastrozole alone 4.8 vs 2.4 months, HR 0.63; 95%CI, 0.47-0.84, p=0.0016) as first-line therapy. [Kaufman et al. 2009] Similarly, in another phase III trial, patients with known ER+/HER2+ tumors (n=219), derived higher benefit from the combination of lapatinib and letrozole as compared to letrozole alone (PFS 8.2 vs 3.0 months, HR 0.71; 95%CI 0.53-0.96; p=0.019). [Johnston et al. 2009]

In a neoadjuvant trial, Chang et al. recently reported that 12 weeks of preoperative trastuzumab plus lapatinib associated with letrozole (+/- goserelin) without chemotherapy can induce pCR in 21% of ER-positive/HER2-positive BC patients. [Chang et al. 2011]

1.3.5 Rationale for the study

Recent evidences suggest that a broaden blockade of HER2 signalling in HER2-positive breast cancer is more effective than a single-blockade and that by combining two anti-her2 agents without chemotherapy promising pCR rates can be achieved. However, extensive crosstalk clearly exists between the ER and HER2 pathways and a critical research question regarding the

treatment of ER+/HER2+ breast cancers include whether chemotherapy can be avoided in a subset of patients, provided both ER and HER2 are simultaneously inhibited. Studies of preoperative endocrine treatment for HR+ disease suggested a predictive role for short-term Ki67 evaluation, indicating that it might be used as a biomarker to identify those patients more likely to respond to preoperative hormonal therapy.

On these premises, this neoadjuvant study will determine the activity of molecular response guided treatment for HER2+/HR+ patients. The potential of short-term Ki67 response to identify those HR+/HER2+ patients likely to respond to dual anti-HER2 blockade combined with hormonal treatment and for whom the addition of chemotherapy could be spared will be investigated.

2. STUDY OBJECTIVES

2.1 Primary objective

- Primary aim is to evaluate the rate of pcR as defined as complete disappearance of invasive tumor in breast and axillary nodes

2.2 Secondary objectives

- To estimate the percentage of clinical objective responses (cOR) (complete plus partial) in the breast (US)
- To estimate the percentage of breast conservative surgery
- To evaluate the safety profile
- Correlative biomarker analyses

3. STUDY PLAN AND PROCEDURES

3.1 Overall study design and flow chart

This is a multicenter, phase II neoadjuvant trial. in hormone-sensitive, HER2 positive, postmenopausal breast cancer patients stage II-IIIA.

10 Italian Centres will participate to this study.

Patients will undergo to a core-biopsy of the primary tumor, for the histological diagnosis and the biological characterization of the tumor.

At least 3-4 cores have to be collected, to allow for routine pathological examinations, immunohistochemical studies and for molecular analysis. At least 1 core of the tumor tissue will be snap-frozen in liquid nitrogen and stored at –80°C, until delivery to the central laboratory. Other cores have to be formalin-fixed, and sent to the local laboratory for diagnostic workup.

Paraffin embedded specimens will be sent (either as block or slices – at least 12) to the central laboratory for centralized review.

In case of clinically or sonographically enlarged axillary lymph-nodes, a fine needle aspiration is recommended, to document the nodal involvement.

Largest tumor diameters will be measured by calliper, by ultrasound and by mammography before and after primary systemic treatment.

The ultrasonographic measure will be used as reference to assess the clinical response.

Surgeon must declare the type of surgical procedure he would apply in case of an upfront surgery.

To rule out patients with metastatic disease, a full staging will be performed with

- Chest radiogram (all the patients)
- Liver ultrasound (all the patients)
- Bone scan (when clinically indicated or in case of positive axillary nodes)
- CT scan when clinically indicated

Hematological exams and routine biochemistry will be performed to assess possible contraindication to treatment. Cardiac function will be evaluated by ECG and by Echocardiogram (or MUGA scan) before systemic treatment.

After confirmation of eligibility and informed consent signature, the patients will start letrozole 2.5 mg p.o. daily for 2-3 weeks. After 2 weeks, a biopsy to assess ki67 inhibition will be performed. Letrozole will be continued until the result of Ki67 (local lab) will be available. At least 3 slides will be sent to the central lab for centralized KI67 re-evaluation.

Patients defined as molecular responders (Ki67 reduction ≥ than 20% from baseline at day 14) will start therapy with the combination of letrozole, trastuzumab and pertuzumab, according to the following schedule:

- Pertuzumab 840 mg loading dose iv on day one, followed by 420 mg iv every 3 weeks for 5 cycles
- Trastuzumab 8 mg/kg loading dose iv on day one, followed by 6 mg/kg iv on day 2 of each subsequent 3 weekly cycle
- Letrozole 2.5 mg daily p.o. for 4 months

If no reactions on cycle 1, pertuzumab and trastuzumab will be administered on the same day for the following 4 cycles.

Patients defined as molecular non-responders (Ki67 reduction less than 20% from baseline at day 14) will stop letrozole and will receive chemotherapy with weekly paclitaxel in combination with trastuzumab and pertuzumab, at the following doses:

- Pertuzumab 840 mg loading dose iv on day one, followed by 420 mg iv every 3 weeks for 5 cycles
- Trastuzumab 8 mg/kg loading dose iv on day 2, followed by 6 mg/kg iv on day 2 of each subsequent 3 weekly cycle
- Paclitaxel 80 mg/sqm iv administered on a weekly basis starting from day 2 for 13 weeks

Standard premedication will be administered prior to each iv infusion.

If no reactions on cycle 1, pertuzumab, trastuzumab and paclitaxel will be administered on the same day (day 1) for the following 4 cycles.

Clinical changes of the primary tumor in the breast will be evaluated every 3 weeks by palpation.

Before surgery, clinical evaluation (by calliper, mammogram and ultrasonography) will be performed. The ultrasonographic measurements will be used to assess the clinical response.

To assess the onset of distant metastases, a chest radiogram and a liver ultrasound will be performed before surgery.

Patients will undergo to surgery within 3 weeks from the last iv therapy. Conservative surgery will be adopted whenever possible, according to local guidelines. Sentinel node biopsy is allowed provided that no evidence of nodal metastases was recorded at initial tumor staging.

Specimens from surgery will be analysed for tumor response and for the assessment of biological parameters. Paraffin embedded specimens will be sent (either as block or slices – at least 12) to the central laboratory.

Treatment after surgery:

Post-operative adjuvant treatments will be administered at discretion of each Center.

Duration of treatment

Both treatments will be administered for five 21-day cycles prior to surgery, that will be performed within 3 weeks from the last iv therapy infusion.

Study treatment will be stopped in case of disease progression, unacceptable toxicity, withdrawal of consent, or death. Patients will be followed until surgery.

3.2 Study Procedures and Assessments

Within 4 weeks prior to first dose

• Subject to sign the informed consent form (ICF)

• Ascertain availability of tumor tissue for biomarker analyses

• Radiologic tumor assessment (mammogram and ultrasound)

• Routine work up staging (liver US, chest XR, bone scan and CT scan when clinically indicated)

• Record any non-serious and serious AEs and assign appropriate toxicity grade

Within 2 weeks prior to first dose

• Physical examination including tumor measurement (by calliper)

• Surgeon's statement of the surgical procedure needed without primary CT

• Medical history, concomitant medications, ECOG Performance Status

• Electrocardiogram (12-lead ECG) ; Echocardiogram or MUGA scan (note that baseline and on treatment scans should be performed using the same modality and preferably at the same institution)

• Vital signs (blood pressure and pulse rate, body temperature, height, and body weight)

• Hematology and blood chemistry

• Record any non-serious and serious AEs and assign appropriate toxicity grade

Pre-dose on Day 1

The following assessments are to be performed within 3 days prior to the first dose of Study therapy

• Physical examination including a review of the inclusion/exclusion criteria. Any changes in the subject's mental or physical condition since the time of screening that would make the subject ineligible for the study should be considered

• Vital signs (blood pressure and pulse rate, body temperature)

• Review baseline signs and symptoms. Any changes from screening will be recorded on the Current Medical Conditions/ Baseline Signs and Symptoms CRF

• Review of screening laboratory results

• Record all medication(s) received within 2 weeks prior to the first dose of investigational product and note if the medication is continuing

• Record any non-serious and serious AEs and assign appropriate toxicity grade

• On Day 1 of each monthly visit, a 21 days supply of letrozole will be dispensed to the patient with instructions for taking the medication. The importance of compliance will be reviewed with the patient.

Every 3 weeks

The following assessments are to be performed every 3 weeks:

• Hematology and blood chemistry

• Physical examination, including tumor measurement (by caliper)

• ECOG Performance Status; Vital signs (blood pressure and pulse rate, body temperature, and body weight)

• Record any non-serious and serious AEs and assign appropriate toxicity grade

• Record all concomitant medication(s) added and/or changed

• A 3-week supply of letrozole will be dispensed to the patient

Assessments at Study Conclusion or Withdrawal from Therapy

The following assessments will be performed at the end of the treatment period and if possible, when a subject is prematurely withdrawn from study for any reason:

• Physical examination, including tumor measurement (by caliper)

• Tumor measurement by mammogram and ultrasonography

• Vital signs (blood pressure and pulse rate, body temperature, and body weight)

• ECOG Performance Status

• Electrocardiogram (12-lead ECG); Echocardiogram or MUGA scan

• Record any AE(s) and serious adverse event(s) (SAEs) and assign appropriate toxicity grade

• Record all concomitant medications(s) added and/or changed

• Hematology and blood chemistry

• Chest XR and liver US

3.3 Laboratory assessment

The total volume of blood loss for laboratory assessments at each visit will be approximately 20 mL. Normal ranges for the study laboratory parameters must be supplied to the Sponsor before the study starts.

Hematology and biochemistry will be done as part of regular safety assessments at screening/baseline, every treatment cycle, and prior to surgery. Assessments must be performed at each cycle within 3 days (with results available) prior to the administration of study medication.

The following parameters will be evaluated:

Hematology

White blood cell count (total); Absolute neutrophil count; Hemoglobin; Platelet count

Biochemistry

Sodium, potassium, calcium, chloride, magnesium, blood urea nitrogen, uric acid, total protein, albumin, ALP, ALT, AST, GGT, LDH, total bilirubin, creatinine, and blood glucose and calculated creatinine clearance at baseline.

Coagulation.

All patients will have INR and aPTT or PTT testing at baseline.

3.4 Biomarkers evaluation

The tumor samples (block or tissue slices) will be centrally collected in a Tumor tissue bank. The following biomarkers will be centrally evaluated: pAKT, pTEN, ki67. Treatment allocation after 2 weeks of will be performed on the basis of local laboratory Ki 67 assessment, and will be centrally reviewed.

Gene mutation analyses will be carried-out to study the occurrence of mutations in the PIK3CA gene. Fresh tumor tissue from core-biopsy must be snap frozen, to perform a microarray analysis of the gene expression profile before treatment and to evaluate its correlation with response.

4. STUDY POPULATION

4.1 Overview

The target population for this study is postmenopausal patients with diagnosis of HER2 positive, hormone receptor positive, stage II-IIIA, previously untreated breast cancer.

4.2 Inclusion criteria

Patients may be included in the study only if they met all the following criteria

- female patients with primary diagnosis of infiltrating breast cancer
- HR positivity (ER ≥ 10% and/or PgR ≥10%) and HER2 positivity (IHC 3+ or FISH/CISH amplification), as assessed by local laboratory.
- Stage II-IIIA
- age >18 yrs
- ECOG Performance Status 0-1
- Postmenopausal status, defined by at least one of the following:

  60 years of age;

  < 60 years of age and amenorrheic for >/=12 months prior to day 1

  < 60 years of age, without a uterus, and luteinizing hormone (LH) and follicle stimulating hormone (FSH) values within postmenopausal range

  Prior bilateral oophorectomy

  Prior radiation castration with amenorrhea for at least 6 months
- Cardiac ejection fraction within the institutional range of normal (as measured by echocardiogram or MUGA scan).
- Normal organ and marrow function as defined below:

  (leukocytes >=3000/mcL;

  absolute neutrophil count >=1,500/mcL;

  platelets >=100,000/mcL;

  total bilirubin within 1.25 x normal institutional limits (with the exception of Gilbert's syndrome);

AST (SGOT)/ALT(SGPT) within 1.25 x institutional upper limit of normal

creatinine within normal institutional limits

- Availability of tumor tissue suitable for biological and molecular examination before starting primary treatment
- Ability to understand and the willingness to sign a written informed consent document

4.3 Exclusion criteria

Patients will be excluded from the study for <u>any</u> of the following reasons

- Stage IIIB, IIIC, and inflammatory breast cancer
- Stage IV breast cancer
- Prior treatment with chemotherapy, endocrine therapy or radiotherapy. Prior treatment with HER2 targeting therapies
- LVEF below the ULN
- Uncontrolled hypertension (systolic >150 mm Hg and/or diastolic >100 mm Hg) or clinically significant (i.e. active) cardiovascular disease: cerebrovascular accident (CVA)/stroke or myocardial infarction within 6 months prior to first study medication, unstable angina, congestive heart failure (CHF) of New York Heart Association (NYHA) grade II or higher, or serious cardiac arrhythmia requiring medication.
- Received any investigational treatment within 4 weeks of study start.
- Subjects with known infection with HIV, HBV, HCV
- Known hypersensitivity to any of the study drugs or excipients.
- Dyspnoea at rest or other disease requiring continuous oxygen therapy.
- Psychiatric illness/social situations that would limit compliance with study requirements
- Subjects assessed by the investigator to be unable or unwilling to comply with the requirements of the protocol

4.4 Other Eligibility Criteria Considerations

To assess any potential impact on subject eligibility with regard to safety, the investigator must refer to the following document(s) for detailed information regarding warnings, precautions, contraindications, adverse events, and other significant data pertaining to the investigational

product(s) being used in this study: pertuzumab Investigator Brochure, trastuzumab Product Information Sheet, Letrozole (Femara) Product Information Sheet, and paclitaxel Product Information Sheet

4.5 Discontinuation of subjects from treatment and assessment

4.5.1 Criteria for discontinuation

Patients have the right to withdraw from the study at any time for any reason.

Patients may be discontinued from study treatment and assessment at any time. Specific reasons for discontinuing a subject from this study are:

1. Voluntary discontinuation by the subjects who are at any time free to discontinue their participation in the study, without prejudice to further therapy
2. Safety reasons as judged by the investigators
3. Severe non-compliance to treatment, as judged by the Investigators
4. Incorrect enrolment or randomization of the subject.
5. Death
6. Subject lost to follow up
7. Objective progression of the disease

4.5.2 Procedures for discontinuation

The reason for discontinuation and the date must be documented on the case report form (CRF). In the case that the patient decides to prematurely discontinue study treatment (“refuses treatment”), she should be asked if she agrees to continue to be followed for post-treatment assessments.

If possible, any investigational product should be returned by the subject.

At discontinuation, all on-going study related toxicities and SAEs must be followed until resolution, unless in the investigators’ opinion the condition is unlikely to resolve, due to the subject’s underlying disease.

After discontinuation from treatment, subjects must be followed up for all the existing and new AEs for 30 days after the last dose of study drug. All new AEs occurring during that period must be recorded and followed up until resolution.

5 TREATMENTS

5.1 Non Investigational products

5.1.1 Letrozole

Letrozole will be obtained directly by the investigational sites for use during this study. For further details, see the applicable Summary of Product Characteristics and local prescribing information.

5.1.2 Trastuzumab

Trastuzumab will be a freeze-dried preparation at a nominal content of either 440 mg or 150 mg per vial. Vial size will also vary by country. For further details, see the applicable trastuzumab Summary of Product Characteristics and local prescribing information.

5.1.3 Paclitaxel

Paclitaxel will be obtained locally by the investigational sites. Refer to the paclitaxel Summary of Product Characteristics for information on formulation, preparation, and administration.

5.2 Investigational products

5.2.1 Pertuzumab

Pertuzumab is provided as a single-use formulation containing 30 mg/mL pertuzumab formulated in 20 mM L-histidine (pH 6.0), 120 mM sucrose, and 0.02% polysorbate 20. Each 20-cc vial contains approximately 420 mg of pertuzumab (14.0 mL/vial). Pertuzumab is intended for use only in clinical trials. For further details, see the pertuzumab Investigator Brochure (IB).

5.3 Doses and treatment regimens

Letrozole:

Letrozole will be prescribed according to the product labelling (2.5 mg orally once daily), for 2 weeks.

<u>After 2 weeks, according to the molecular response, patients will be subdivided in two groups:</u>

A) Molecular responders

These patients will continue letrozole 2.5 mg orally once daily. Patients will start therapy with pertuzumab and trastuzumab according to the following doses-schedules:

Pertuzumab:

Pertuzumab will be administered as an intravenous infusion on Day 1 of the first treatment cycle as a loading dose of 840 mg. The following 4 courses will be administered at the dose of 420 mg on Day 1 every 3 weeks.

Initial infusions of pertuzumab will be administered over 60 (± 10) minutes and patients observed for 60 minutes from the end of infusion for infusion-related symptoms such as fever, chills, hypotension, shortness of breath, skin rash, headache, nausea and/or vomiting. Interruption or slowing of the infusion may reduce such symptoms. If the infusion is well tolerated, subsequent infusions may be administered over 30 to 60 (± 10) minutes, with patients observed for a further 30 minutes.

Trastuzumab:

Trastuzumab will be administered as an intravenous infusion on day 2 of the first treatment cycle as a loading dose of 8 mg/kg, followed by 6 mg/kg on day 2 of each subsequent 3 weekly cycle; to be administered in line with product labelling.

If no reactions on cycle 1, pertuzumab and trastuzumab will be administered on the same day for the following 4 cycles.

B) Molecular non-responders

These patients will stop letrozole. Patients will start therapy with pertuzumab, trastuzumab and paclitaxel according to the following doses-schedules:

Pertuzumab:

Pertuzumab will be administered as an intravenous infusion on Day 1 of the first treatment cycle as a loading dose of 840 mg. The following 4 courses will be administered at the dose of 420 mg on Day 1 every 3 weeks.

Initial infusions of pertuzumab will be administered over 60 (± 10) minutes and patients observed for 60 minutes from the end of infusion for infusion-related symptoms such as fever, chills, hypotension, shortness of breath, skin rash, headache, nausea and/or vomiting.

Interruption or slowing of the infusion may reduce such symptoms. If the infusion is well tolerated, subsequent infusions may be administered over 30 to 60 (± 10) minutes, with patients observed for a further 30 minutes.

Trastuzumab:

Trastuzumab will be administered as an intravenous infusion on day 2 of the first treatment cycle as a loading dose of 8 mg/kg, followed by 6 mg/kg on day 2 of each subsequent 3 weekly cycle; to be administered in line with product labeling.

Paclitaxel:

Paclitaxel will be administered at the dose of 80 mg/sqm weekly for 13 weeks. Paclitaxel will be infused as 1 hour iv infusion on day 2 (with standard premedication).

If no reactions on cycle 1, pertuzumab, trastuzumab and paclitaxel will be administered on the same day for the following 4 cycles.

5.4 Dose modifications, interruptions or delays

5.4.1 Letrozole

There will be no dose reductions of letrozole. If a subject is required to stop treatment with letrozole for more than 3 weeks, the Principal Investigator must be consul If a subject vomits after taking therapy, the subject should be instructed not to retake the dose. Subjects should take the next scheduled dose of therapy. If vomiting persists, then the subject should contact the investigator.

5.4.2 Paclitaxel

Before each paclitaxel administration, hematological parameters and biochemistry have to be controlled.

Full dosage of chemotherapy drugs will be delivered if WBC is $\geq$ 2,5 X 10^9/L, ANC is $\geq$ 1.0 $\times$ 10^9/L and platelets $\geq$ 100 $\times$ 10^9/L, and the patient has recovered below grade 2 for all non hematological toxicity.

A one week delay will be adopted for lower values, until a maximum of 3 weeks.

In case of grade 4 haematological toxicity lasting more than 3 (ANC) or 5 (PLT) days during therapy, next courses will be delivered at 75% of the projected dose. Prophylactic

administration of G-CSF is not allowed. G-CSF use can be used as rescue for grade 4 ANC toxicity lasting more than 3 days, or in case of neutropenic fever. In case of sensory-motor neurological toxicity ≥ Grade 2, paclitaxel will be discontinued until recovery. Chemotherapy will be permanently discontinued in case of non hematological toxicity grade 4. For grade 3 non-hematological toxicity, a case by case evaluation has to be made by the investigator. Doses which have been reduced for toxicity must not be re-escalated.

5.4.3 Trastuzumab and Pertuzumab

Dose reductions are not permitted for toxicity. Administration of trastuzumab and pertuzumab may be delayed to assess or treat adverse events.

If the patient misses a dose of pertuzumab for a cycle 1 or more (i.e., the 2 sequential administration times are 6 weeks or more apart), a re-loading dose of pertuzumab (840 mg) should be given as described in the product labeling. Subsequent maintenance pertuzumab doses of 420 mg will then be given every 3 weeks, starting 3 weeks later. If the patient misses a dose of trastuzumab by more than 1 week, reloading of trastuzumab should follow approved local Product Information. Subsequent maintenance trastuzumab doses of 6 mg/kg will then be given every 3 weeks, starting 3 weeks later.

5.4.3.1 Cardiac safety

For trastuzumab and pertuzumab, there is no dose adjustment foreseen. The infusions will be stopped completely according to the following:

- Development of NYHA class III/IV cardiac dysfunction
- Grade 3-4 non hematologic toxicity, until recovery to grade <2

For asymptomatic patients with drop in the LVEF, refer to the following algorithm:

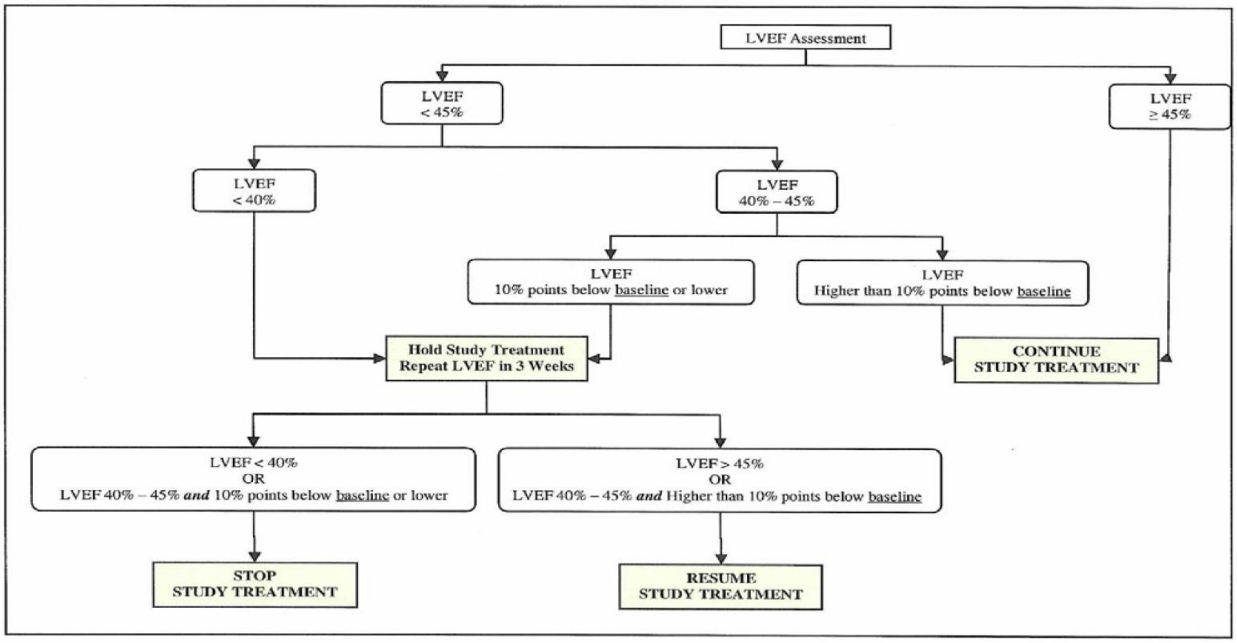

5.4.3.2 Infusion-Associated Symptoms and Allergic Reactions

Administration of monoclonal antibodies, including pertuzumab and trastuzumab, may cause infusion-associated symptoms such as fever, chills, hypotension, shortness of breath, skin rashes, headache, nausea, vomiting, or allergic reactions. Careful consideration must be made before enrolling patients with chronic lung disease into the study.

Study treatment will be administered in a setting with emergency equipment and staff that is trained to monitor for and respond to medical emergencies. Patients who experience an NCI-CTCAE Grade 4 allergic reaction, acute respiratory distress syndrome (ARDS), or bronchospasm will be discontinued from study treatment.

Patients who experience infusion-associated symptoms may be managed by:

- Slowing or stopping the or pertuzumab infusion

- Supportive care with oxygen, beta-agonists, antihistamines, antipyretics, or corticosteroids as appropriate at the investigator's discretion

- Premedication with corticosteroids, antihistamines, and antipyretics may be used before subsequent pertuzumab infusions at the investigator’s discretion.

If infusion-associated symptoms occur, patients will be monitored until complete resolution of signs and symptoms.

Patients who experience trastuzumab SC injection-related symptoms may be pre-medicated with paracetamol and antihistamines for subsequent injections.

5.4.3.3 Incomplete Loading Dose

In case the whole loading dose of pertuzumab cannot be administered due to an infusion reaction or other reason, the following guidelines apply:

The patient should receive at least 50% of the loading dose in the first week. Therefore, if the patient receives less than 50% of the Cycle 1 dose, the patient should receive the remainder before Day 22, preferably within the first week. Thereafter, the patient should receive the usual maintenance dose 3 weeks after the first interrupted dose, as routinely scheduled. For example, if a patient received only approximately 50% of the scheduled loading dose (i.e., only 4 mg/kg instead of 8 mg/kg of trastuzumab; or only 420 mg instead preferably in the first week, and then regular maintenance doses (6 mg/kg of trastuzumab; 420 mg of pertuzumab) on Day 22, as routinely scheduled. If the patient receives between 50-75% of the dose, the patient should receive the remainder before Day 22, preferably within the first two weeks of Cycle 1. For example, if a patient received only approximately 60% of the scheduled loading dose, the patient should receive the remaining 40%, within 2 weeks after the interrupted loading dose. Thereafter, the patient should receive the regular maintenance doses on Day 22, as routinely scheduled. If the patient received ≥ 75% of the loading dose, additional loading is probably not necessary. However, the remainder of the loading dose may be given at the investigator's discretion. In such a case, the remainder may be given at any time before the next scheduled dose or the patient may be given an additional loading dose on Day 22. If, after receiving an incomplete loading dose on Day 1, the patient cannot attend the site until Day 22, the patient should receive a second loading dose on Day 22. However, every effort should be made to give the remainder of the dose prior to Day 22.

5.4.3.4 toxicities attributable to EGFR inhibition

Although pertuzumab targets HER2, because of its role in heterodimerization with other members of the HER family (e.g., EGFR), it may cause toxicities associated with the use of EGFR tyrosine kinase inhibitors. Diarrhea has been observed in approximately 50% of patients being

treated with pertuzumab in Phase II single-agent studies, and up to 70% of patients in combination therapy studies, and was NCI-CTCAE Grade 1 or 2 in the majority of cases. For patients experiencing diarrhea, early intervention with loperamide should be considered. Rash has also been observed with EGFR tyrosine kinase inhibitors. The rash was generally mild to moderate in intensity and appeared to be treatable in some patients with standard acne therapies, including topical and oral antibiotics.

5.4.3.5 Actions to be Taken in Case of Pertuzumab and Trastuzumab Related Toxicity

toxicity	Action
Non-hematological, Grade 1 or 2 (NCI-CTCAE; excluding cardiac*) toxicity	Continue with study treatment
Non-hematological, Grade 3 or 4 (NCI-CTCAE; excluding cardiac*) toxicity	Hold study treatment (all medication in the cycle) until recovery to Grade ≤ 2.
Recurrence of non-hematological, Grade 3 or 4 (NCI-CTCAE; excluding cardiac*) toxicity upon re-challenge	Discontinue the related study medication (pertuzumab or trastuzumab) permanently. Continue treatment as deemed suitable by local investigator.
Cardiac toxicity (asymptomatic drop in LVEF or symptomatic congestive heart failure)	Study treatment (all medication in the cycle) to be held, continued or resumed according to the algorithm depicted in section 5.4.3.1 Related study medication (pertuzumab or trastuzumab) to be discontinued permanently in case of symptomatic CHF
Cardiac toxicity (NCI-CTCAE; other cardiac toxicities not covered by treatment algorithm in section 5.4.3.1)	Actions must follow rules 1. to 3. for non-hematological toxicities
Hematological toxicity – Neutropenia	Hold study treatment (all medication in the cycle) until neutrophils ≥ 1.0 x 10^9/L.

5.5 Storage and Accountability

It is the investigator/institution's responsibility to establish a system for storage and handling study treatments, including investigational products, so as to ensure that:

- The deliveries of such products are correctly received by a responsible person (e.g., a pharmacist)
- Deliveries are recorded
- Study treatments are handled and stored safely and properly
- Study treatments are dispensed only to study subjects in accordance with the protocol

At the end of the study, it must be possible to reconcile delivery records with records of usage and destroyed/returned stock. Any discrepancy must be accounted for. Certificates of delivery and return must be signed, preferably by the investigators or a pharmacist.

5.6 Pre-study, concomitant, and post-study treatment(s)

All protocol-allowed medications taken by the patient for concomitant disease should continue as necessary during the study and be recorded on the electronic case report form (eCRF). Treatments prescribed to patients should be adapted according to the local standard of care practice. The following treatments/procedures are permitted:

- Paracetamol (acetaminophen) or other analgesics, and diphenhydramine,
- chlorpheniramine, or other antihistamines can be used according to local clinical practice for the prevention and treatment of infusion reactions associated with pertuzumab and/or trastuzumab
- Medication to treat diarrhea (e.g., loperamide)
- Steroids, antihistamines, and H2-receptor antagonists for paclitaxel premedication according to routine practice at each clinical site
- Inhaled steroids for asthma

<u>The following treatments are not permitted:</u>

- Treatment with other systemic anticancer agents (e.g., chemotherapy, hormonal therapy, immunotherapy) or other treatments not part of protocol specified anticancer therapy
- Any oral, injected or implanted hormonal methods of contraception

- Concurrent investigational agents of any type
- Initiation of herbal remedies for cancer treatment. Herbal remedies initiated prior to study entry and continuing during the study are permitted and must be reported on the appropriate eCRF

The following treatments should be avoided because of the risk of immunosuppression:

- Chronic or high-dose oral corticosteroid therapy
- Tumor necrosis factor-α inhibitors
- Anti-T cell antibodies

5.7 Treatment compliance

Treatment compliance for letrozole will be monitored by pill count every 3 weeks.

Lack of treatment compliance will not led to discontinuation in this study.

6. SAFETY ASSESSMENT

6.1. General safety assessment

Patients will be assessed by prior medical history, vital signs (including blood pressure, heart rate, temperature), weight and height (screening only), physical examination, adverse events and concomitant medications. A complete medical history (including demographic profile and prior treatments for cancer) will be documented at screening.

A general physical exam will be performed at every cycle. SC injection sites will be checked at every visit and blood pressure and heart rate will be measured before treatment at each cycle.

Adverse events will be monitored and documented continuously during study. Serious adverse events (SAEs) will also be monitored, documented and reported; refer to Sections 6.5 for details on SAE reporting and follow-up requirements. All AEs and SAEs (including patients' symptoms and signs of toxicity and clinically significant haematological and biochemical parameters) will be graded according to National Cancer Institute (NCI) Common Terminology Criteria for Adverse Events (CTCAE) version 4.0. Changes in concomitant medication will be recorded at each study visit.

The investigator is responsible for the detection and documentation of events meeting the criteria and definition of an AE or SAE, as provided in this protocol. During the study when there is a safety evaluation, the Investigator or site staff will be responsible for detecting, documenting and reporting AEs and SAEs, as detailed in both this section of the protocol and in the AE/SAE section of the CRF.

AEs not listed on the CTCAE should be graded as follows:

CTC Grade	Equivalent To:	Definition
Grade 1	Mild	Discomfort noticed but no disruption of normal daily activity.
Grade 2	Moderate	Discomfort sufficient to reduce or affect daily activity; no treatment or medical intervention is indicated although this could improve the overall well-being or symptoms of the patient.
Grade 3	Severe	Inability to work or perform normal daily activity; treatment or medical intervention is indicated in order to improve the overall well-being or symptoms; delaying the onset of treatment is not putting the survival of the patient at direct risk.
Grade 4	Life-threatening/ disabling	An immediate threat to life or leading to a permanent mental or physical conditions that prevents work or performing normal daily activities; treatment or medical intervention is required in order to maintain survival.
Grade 5	Death	AE resulting in death.

6.2 Drug – Adverse Event relationship

The causality relationship of study drug to the AE will be assessed by the investigator as either:

Yes or No

If there is a reasonable suspected causal relationship to the study medication, i.e. there are facts (evidence) or arguments to suggest a causal relationship, drug-event relationship should be assessed as **Yes**.

The following criteria should be considered in order to assess the relationship as **Yes:**

- Reasonable temporal association with drug administration.
- It may or may not have been produced by the patient's clinical state, environmental or toxic factors, or other modes of therapy administered to the patient.
- Known response pattern to suspected drug.
- Disappears or decreases on cessation or reduction in dose.
- Reappears on rechallenge.

The following criteria should be considered in order to assess the relationship as **No**:

• It does not follow a reasonable temporal sequence from administration of the drug.

• It may readily have been produced by the patient's clinical state, environmental or

toxic factors, or other modes of therapy administered to the patient.

• It does not follow a known pattern of response to the suspected drug.

• It does not reappear or worsen when the drug is readministered.

6.3 Cardiac safety assessment

Cardiac function will be evaluated by measuring LVEF using echocardiography or MUGA scan (method selected according to local practice), ECG, and assessment of cardiac signs and symptoms.

LVEF measurement and ECG will be performed at screening and at the completion of the 5 treatment cycles, before surgery. Assessment of cardiac signs and symptoms will be performed at every cycles. Additional LVEF measurement /ECG will be repeated if clinically indicated.

Any patient who develops clinical signs or symptoms suspicious of cardiac failure at any time during the study should undergo an LVEF assessment immediately.

Symptomatic left ventricular dysfunction (congestive heart failure) will be graded according to NCI-CTCAE version 4.0 and the New York Heart Association (NYHA) functional classification

Patients whose LVEF falls ≥ 10 percentage points from screening and to a LVEF < 50% may require temporary or permanent cessation of trastuzumab and pertuzumab.

A repeat LVEF assessment should be performed approximately 3 weeks later. If the LVEF has not improved or has declined further, trastuzumab and pertuzumab should be discontinued. All such patients should be referred for assessment by a cardiologist and followed up. Trastuzumab and pertuzumab should also be discontinued in any patient who develops clinically significant heart failure

6.4 Safety parameters and definition

Safety assessments will consist of monitoring and recording adverse events, including serious adverse events and non-serious adverse events of special interest; measurement of protocol-specified safety laboratory assessments; measurement of protocol-specified vital signs; and other protocol-specified tests that are deemed critical to the safety evaluation of the study. Certain types of events require immediate reporting to the Sponsor, as outlined in Section 6.5.

6.4.1 Adverse Events (AE)

According to the ICH guideline for Good Clinical Practice, an adverse event (AE) is any untoward medical occurrence in a clinical investigation subject administered a pharmaceutical product, regardless of causal attribution. An adverse event can therefore be any of the following:

• Any unfavourable and unintended sign (including an abnormal laboratory finding), symptom, or disease temporally associated with the use of a medicinal product, whether or not considered related to the medicinal product

• Any new disease or exacerbation of an existing disease (a worsening in the character, frequency, or severity of a known condition)

• Recurrence of an intermittent medical condition (e.g., headache) not present at baseline

• Any deterioration in a laboratory value or other clinical test (e.g., ECG, X-ray) that is associated with symptoms or leads to a change in study treatment or concomitant treatment or discontinuation from study drug

• Adverse events that are related to a protocol-mandated intervention, including those that occur prior to assignment of study treatment (e.g., screening invasive procedures such as biopsies).

6.4.2 Laboratory Test Abnormalities

Local laboratories will be used for all safety laboratory tests. Laboratory test reports should be included in the patient chart and made available for routine monitoring and source document verification.

Any treatment-emergent abnormal laboratory result that is clinically significant should be recorded as a single diagnosis on the AE page in the eCRF. Clinical significance is defined as meeting one or more of the following conditions:

• Accompanied by clinical symptoms

• Leading to a change in study medication (e.g. dose modification, interruption or permanent discontinuation)

• Requiring a change in concomitant therapy (e.g. addition of, interruption of, discontinuation of, or any other change in a concomitant medication, therapy or treatment).

Laboratory test value abnormalities that are not considered clinically significant should not be recorded as AEs in the eCRF.

Any laboratory result abnormality fulfilling the criteria for an SAE should be reported as such, in addition to being recorded as an AE in the eCRF.

6.4.3 Serious Adverse Events (SAEs)

An SAE is an experience that suggests a significant hazard, contraindication, side effect or precaution. It is any AE that at any dose fulfils at least one of the following criteria:

- Fatal (i.e., the adverse event actually causes or leads to death)
- Life threatening (i.e., the adverse event, in the view of the investigator, places the patient at immediate risk of death)
- This does not include any adverse event that had it occurred in a more severe form or was allowed to continue might have caused death.
- Requires or prolongs inpatient hospitalization
- Results in persistent or significant disability/incapacity (i.e., the adverse event results in substantial disruption of the patient's ability to conduct normal life functions)
- Congenital anomaly/birth defect in a neonate/infant born to a mother exposed to study drug
- Significant medical event in the investigator's judgment (e.g., may jeopardize the patient or may require medical/surgical intervention to prevent one of the outcomes listed above)

**The term sudden death should be used only when the cause is of a cardiac origin as per standard definition. The terms death and sudden death are clearly distinct and must not be used interchangeably.

The terms "severe" and "serious" are not synonymous. Severity refers to the intensity of an adverse event (rated as mild, moderate, or severe, or according to NCI CTCAE criteria; see Section 5.3.3); the event itself may be of relatively minor medical significance (such as severe headache without any further findings). Severity and seriousness need to be independently assessed for each adverse event recorded on the eCRF. Serious adverse events are required to be reported by the investigator to the Sponsor within 24 hours after learning of the event (see Section 6.5 for reporting instructions).

6.5 Handling of Safety Parameters

6.5.1 Reporting of Adverse Events

All adverse events (related and unrelated) occurring during the study and up to 28 days after the last dose of study medication must be reported on the eCRF.

6.5.2 Reporting of Serious Adverse Events (Immediately Reportable)

Any clinical adverse event or abnormal laboratory test value that is *serious* and which occurs during the course of the study , must be reported to the Sponsor **within *24 hours*** of the investigator becoming aware of the event (expedited reporting).

Related Serious Adverse Events ***MUST*** be collected and reported regardless of the time elapsed from the last study drug administration, even if the study has been closed. Unrelated Serious Adverse Events must be collected and reported during the study and for up to 28 days after the last dose of study medication. The study will comply with all local regulatory requirements. The definition and reporting requirements of ICH Guideline for Clinical Safety Data Management, Definitions and Standards for Expedited Reporting, Topic E2 will be adhered to.

6.5.3 Reporting of Non-Serious Adverse Events of special interest

Symptomatic Left Ventricular Systolic Dysfunction

Symptomatic left ventricular systolic dysfunction should be reported with the event term "Congestive heart failure" Signs and symptoms should however be entered in the comments part of the AE page in the eCRF. Congestive heart failure should be graded according to NCI-CTCAE v 4.0 and according to the NYHA classification.

Symptomatic congestive heart failure must be reported irrespective of causal relationship during the full course of the study.

Asymptomatic Left Ventricular Systolic Dysfunction

Asymptomatic declines in LVEF should not be reported as AEs since LVEF data are collected separately in the eCRF. Exceptions to this rule are as follows:

- Any asymptomatic decline in LVEF requiring treatment or leading to withholding/discontinuation of trastuzumab SC must be reported as an AE. In this case the

event should be reported with the event term "left ventricular systolic dysfunction" and graded according to NCI-CTCAE v 4.0 (Appendix 6).

The following table summarizes the reporting conventions for left ventricular systolic dysfunction.

**Reporting Conventions for Left Ventricular Systolic Dysfunction Observation**	**How to report**	**Term to be reported**	**Grading**
Asymptomatic decline in LVEF	no AE	-	-
Asymptomatic decline in LVEF requiring treatment or leading to withholding/ discontinuation of trastuzumab SC	AE<br>(and SAE report if applicable)	LVSD	NCI CTCAE for LVSD
Symptomatic left ventricular systolic dysfunction	AE<br>and SAE report	Congestive HF	NCI CTCAE for LVSD and NYHA
Abbreviations: AE: adverse event; HF: heart failure; LVEF: left ventricular ejection fraction; LVSD: left ventricular systolic dysfunction; NCI CTCAE: National Cancer Institute Common Terminology Criteria for Adverse Events; NYHA: New York Heart Association; SAE: serious adverse event			

6.5.4 Progression of Underlying Malignancy

Progression of the primary tumor is not reported as an adverse event if it is clearly consistent with the suspected progression of the underlying cancer. Progression of the primary tumor as defined by clinical increase of primary tumor or evidence of metastasis requires immediate discontinuation from study medication and local practice should be followed such as: second-line cytotoxic regimen, surgery and or radiotherapy. Hospitalization due solely to the progression of underlying malignancy should NOT be reported as a serious adverse event.

6.5.5 Follow-up of AEs

The terminal half-life of trastuzumab, derived from population PK analysis of 3 studies in patients with metastatic breast cancer and one trial in patients with NSCLC, is approximately 28 days. It is expected that the terminal half-life of pertuzumab, derived using similar methods, would be similar. Therefore, pertuzumab and trastuzumab may be present in the circulation for 20 weeks (range 18-24) weeks after the last treatment.

After **28 days** continue to follow up AEs as follows:

Related AEs: Follow until one of the following occurs:

– Resolved or improved to baseline

– Relationship is reassessed as unrelated

– Death

– Start of new anti-cancer regimen

– Investigator confirms that no further improvement can be expected

– Clinical or safety data will no longer be collected, or final database closure

Unrelated severe or life threatening AEs: Follow until one of the following occurs:

– Resolved or improved to baseline

– Severity improved to Grade 2

– Death

– Start of new anti-cancer regimen

– Investigator confirms that no further improvement can be expected

– Clinical or safety data will no longer be collected, or final database closure

Unrelated Grade 1 or Grade 2 AEs: Follow until **final safety visit.**

The final outcome of each adverse event must be recorded on the eCRF

7. STATISTICAL SECTION, MEASUREMENTS OF STUDY VARIABLES , DATA COLLECTION AND MANAGEMENT

7.1 Sample Size

Sample size has been estimated by using the optimal Simon's two-stage design. Assuming in Ki67 responders a pCR rate of < 10% with endocrine therapy alone, and of around 15% with dual antiHer2 therapy (pertuzumab + trastuzumab, without endocrine therapy), the combination of endocrine therapy + pertuzumab + trastuzumab will be considered worthwhile in case of a pCR rate= 25%.

Suppose that we want to test

H0 : 0.10

H1: 0.25

- With $\alpha = 0.05$ and $\beta = 0.10$ (power = 0.90), in stage I, n 18 patients are accrued. If </= 2 pCR are observed, then the trial is stopped for futility.
- Otherwise additional n =25 patients are accrued, for a total of 43 patients. If at least 8 pCRs are observed, further investigation (i.e, Phase III trial) is warranted

Assuming that approximately 70% of the patients will have molecular response, a total of 68 patients will be included to allow 48 patients in the hormonal therapy arm, with 10% drop off. The patients not having a molecular response, thus receiving paclitaxel plus trastuzumab-pertuzumab, will be followed and the pCR rate evaluated as internal control.

7.2 Measurements of study variables

7.2.1 Clinical Response

The Clinical Response will be defined according to RECIST criteria comparing the tumor size (largest tumor diameter) before and after treatment, as assessed by ultrasound examination.

7.2.2 Pathologic Response

A pathologic complete response (pCR) is defined the complete absence of infiltrating tumor cells in the breast and in lymph nodes. Residual in situ disease (DCIS) will be included in the pCR category.

7.2.3 Rate of Conservative Surgery

The rate of conservative surgery will be calculated as the percent of the conservative procedures performed after primary therapy. The proportion of patients achieving breast conserving surgery (BCS) will be tabulated. The conversion from mastectomy to BCS will be calculated as the percent difference between the number of conservative surgical procedures feasible at study entry and the number of the conservative procedures performed after primary therapy

7.2.4 Safety measurements and variables

To assess the safety profile, nature, incidence and severity of adverse events (AEs) and serious adverse events (SAEs) will be collected. Incidence of and reasons for study drug dose interruption or reduction and discontinuation will be collected. Toxicities will be graded using NCI Common Terminology Criteria for Adverse Events (CTCAE) version 4.0.

All patients who received at least one dose of treatment will be included in the safety evaluation.

7.2.5 Biomarkers Evaluation

It is the responsibility of the investigator, or a person designated by the investigator, to obtain written informed consent from each individual who has consented to have their samples stored for future research in the 15 years after adequate explanation of the aims, methods, objectives and potential hazards. Patients must receive an explanation that they are completely free to refuse long-term storage of their samples for future research and may withdraw his/ her sample at any time and for any reason during the 15 year storage period of the specimen(s).

7.3 Data collection and management, quality assurance

Data for this study will be recorded via an Electronic Data Capture (EDC) system using electronic Case Report Forms. On eCRFs or other documents submitted to the sponsor, patients should not be identified by their names, but by an identification code.

The data will be transcribed by the site from the paper source documents onto the eCRF. Accurate and reliable data collection will be assured by verification and cross–check of the eCRFs against the investigator's records by the study monitor (source document verification.

For classification purposes, preferred terms will be assigned by the sponsor to the original terms entered on the eCRF, using the most up to date version of MedDRA (medical dictionary for regulatory activities terminology) for adverse events and diseases and the INN (international non-proprietary name) drug terms and procedures dictionary for treatments and surgical and medical procedures.

Monitoring:

Start-up visit: after the local ECs approval, a study monitor from the coordinating centre will visit the study sites before first study subject entry

Monitoring during study: the study monitor will visit each centre after the randomization of the first patient, and thereafter will have regular contacts with the investigational sites, including visits to:

- provide information and support to the investigator(s)
- confirm the adherence to the protocol, verify the accurately record of the data in the eCRFs, verify the correct investigational product's accountability
- perform source data verification, with direct access to all original records for each subject

The monitor will be available between visits if the investigator(s) or other staff at the centres need information and advice.

7.4 Changes to the protocol

If necessary for the protocol to be amended, the amendment and or a new version the study protocol (Amended Protocol) must be notified and approved by ECs.

8. ETHICS

8.1 Ethics review

The final study protocol, including the final version of the Written Informed Consent Form, must be approved by the ECs. The PI is responsible for informing the ECs of any amendment to the protocol in accordance with local requirements.

8.2 Ethical conduct of the study

The study will be performed in accordance with ethical principles that have their origin in the Declaration of Helsinki and are consistent with ICH/GCP

8.3 Written informed consent

The PI at each centre will ensure that the subject is given full and adequate oral and written information about the nature, purpose, possible risk and benefit of the study. Subjects must also be notified that they are free to discontinue from the study at any time. The subject's signed and dated informed consent must be obtained prior to conduct any procedure specific for the study. The original signed Written Informed Consent Form must be stored, and a copy must be given to the patient.

8.4 Subject data protection

The Written Informed Consent Form will explain that the study data will be stored in a computer database, maintaining confidentiality in accordance with national data legislation. All computer data will be identified by an indentification code only.

The investigator must assure that patients' anonymity will be maintained and that their identities are protected from unauthorized parties. On eCRFs or other documents submitted to the sponsor, patients should not be identified by their names, but by an identification code The investigator should keep a patient enrollment log showing codes, names and addresses. The investigator should maintain documents not for submission to The Sponsor, e.g., patients' written consent forms, in strict confidence.

9. STUDY CONTACT FOR PROTOCOL-RELATED INQUIRIES

Dr Valentina Guarneri

Oncology Unit – IOV-IRCCS - University of Padova

Tel: 049 8215291, Email: valentina.guarneri@unipd.it

10. EMERGENCY PROCEDURES

10.1 Procedures in case of medical emergency

The principal investigator(s) at each centre is responsible for ensuring that procedures and expertise are available to handle medical emergencies during the study.

10.2 Procedures in case of overdose

There is currently no known antidote for letrozole. The treatment of AEs associated with overdose should be supportive for the underlying adverse symptoms. To date, no subject has experienced an overdose with these drugs.

Doses of study treatment in excess of that specified in the clinical study protocol are considered to be an overdose. Signs or symptoms of an overdose that meet the criteria of serious should be reported as an SAE in the appropriate timeframes and be documented as clinical sequelae to an overdose

11. USE OF DATA AND PUBLICATIONS POLICY

When all planned patients have completed the study period, the Coordinating Centre will verify the collected data and lock the data base to carry out the analyses.

The statistician will prepare a report that will be discussed with the study coordinator and the principal investigator. On the basis of this report, the principal investigator will prepare a draft article for publication that will be discussed with all involved investigators. The final article, reporting the results of the trial, must be approved by all co-authors before being submitted. Those who have contributed to the protocol and/or to the implementation of the study and/or who have intellectually participated in the drafting of the scientific paper will be considered for the authorship.

The ownership of trial data belongs to the sponsor that can not prevent the publication of the study results.

12. RISKS AND BENEFITS OF PARTICIPATING PATIENTS

The standard treatment of HER2 positive breast cancer consists of chemotherapy combined with trastuzumab. New strategies based on dual anti-Her2 blockade are consistently showing higher activity as compared to single anti-HER2 blockade. However, the expression of hormone receptor, even in the presence of HER2 expression, is associated with a lower rate of pathologic response. Moreover, the combination of hormonal therapy plus anti-Her2 agents has shown interesting level of activity in both advanced and early disease.

This trial is aiming to personalized treatment on the basis of the initial response to endocrine therapy. In particular, this trial is going to evaluate whether, in patient selected on the basis of endocrine responsiveness, the treatment with trastuzumab, pertuzumab and endocrine therapy (without chemotherapy), might result in a pathologic complete remission rate similar to that achievable with chemotherapy and trastuzumab. This would allow to spare the toxicity of chemotherapy. The patients not considered endocrine responsive would receive treatment with chemotherapy and pertuzumab and trastuzumab. The dual anti-Her2 blockade is not associated with increased toxicity as compared to chemotherapy and trastuzumab, which is the standard therapy in this patient population.

The patients are going to receive one additional breast core biopsy, with related discomfort (mainly transient pain).

13. REFERENCES

1. The ATAC (Arimidex, Tamoxifen Alone or in Combination) Trialists' Group. Anastrozole alone or in combination with tamoxifen versus tamoxifen alone for adjuvant treatment of postmenopausal women with early breast cancer: first results of the ATAC randomised trial. Lancet 2002;359:2131–39

2. Attard G, Kitzen J, Blagden SP, et al. A phase Ib study of pertuzumab, a recombinant humanised antibody to HER2, and docetaxel in patients with advanced solid tumours. Br J Cancer 2007;97:1338-43.

3. Baselga J, Semiglazov V, van Dam P, et al. Phase II randomized study of neoadjuvant everolimus plus letrozole compared with placebo plus letrozole in patients with estrogen receptor-positive breast cancer. J Clin Oncol 2009;27:2630-7.

4. Baselga J, Gelmon KA, Verma S, et al. Phase II trial of pertuzumab and trastuzumab in patients with human epidermal growth factor receptor 2-positive metastatic breast cancer that progressed during prior trastuzumab therapy. J Clin Oncol 2010;28:1138-44.

5. Baselga J, Cortés J, Kim SB, et al. Pertuzumab plus trastuzumab plus docetaxel for metastatic breast cancer. N Engl J Med 2012;366:109-19.

6. Baselga J, Bradbury I, Eidtmann H, et al. Lapatinib with trastuzumab for HER2-positive early breast cancer (NeoALTTO): a randomised, open-label, multicentre, phase 3 trial. Lancet 2012;379:633-40.

7. Blackwell KL, Burstein HJ, Storniolo AM, et al. Overall survival benefit with lapatinib in combination with trastuzumab for patients with human epidermal growth factor receptor 2-positive metastatic breast cancer: final results from the EGF104900 Study. J Clin Oncol 2012;30:2585-92.

8. Buzdar AU, Ibrahim NK, Francis D, et al. Significantly higher pathologic complete remission rate after neoadjuvant therapy with trastuzumab, paclitaxel, and epirubicin chemotherapy: results of a randomized trial in human epidermal growth factor receptor 2-positive operable breast cancer. J Clin Oncol 2005;23:3676-85.

9. Chang JCN, Forero-Torres A, Nanda R, et al. TBCRC 006: a multicenter phase II study of neoadjuvant lapatinib and trastuzumab in patients with HER2-overexpressing breast cancer. J Clin Oncol 2011;29(Suppl):Abstract 505.

10. Coombes RC, Hall E, Gibson LJ, et al. A randomized trial of exemestane after two to three years of tamoxifen therapy in postmenopausal women with primary breast cancer. N Engl J Med 2004;3501081-92.

11. Cortés J, Baselga J, Petrella T, et al. Pertuzumab monotherapy following trastuzumab-based treatment: Activity and tolerability in patients with advanced HER2-positive breast cancer. Presented at American Society of Clinical oncology Annual Meeting 2009; Abstract 1022.

12. Dawood S, Broglio K, Buzdar AU, Hortobagyi GN, Giordano SH. Prognosis of women with metastatic breast cancer by HER2 status and trastuzumab treatment: an institutional-based review. J Clin Oncol 2010;28:92-8.

13. Dowsett M, Ebbs SR, Dixon JM, et al. Biomarker Changes During Neoadjuvant Anastrozole, Tamoxifen, or the Combination: Influence of Hormonal Status and HER-2 in Breast Cancer - A Study from the IMPACT Trialists. J Clin Oncol 2005;23:2477-2492.

14. Dowsett M, Smith IE, Ebbs SR, et al. Prognostic value of Ki67 expression after short-term presurgical endocrine therapy for primary breast cancer. J Natl Cancer Inst 2007; 99:167-70

15. Eiermann W, Paepke S, Appfelstaedt J, et al. Preoperative treatment of postmenopausal breast cancer patients with letrozole: A randomized double-blind multicenter study. Ann Oncol 2001;12:527-32

16. Ellis MJ, Coop A, Singht B, et al. Letrozole is more effective neoadjuvant endocrine therapy than tamoxifen for ErbB-1- and/or ErbB-2-positive, estrogen receptorpositive primary breast cancer: evidence from a phase III randomized trial. J Clin Oncol 2001;9:3808-16.

17. Franklin MC, Carey KD, Vajdos FF, Leahy DJ, de Vos AM, Sliwkowski MX. Insights into ErbB signaling from the structure of the ErbB2-pertuzumab complex. Cancer Cell 2004;5:317-28.

18. Gianni L, Lladó A, Bianchi G, et al. Open-label, phase II, multicenter, randomized study of the efficacy and safety of two dose levels of Pertuzumab, a human epidermal growth factor receptor 2 dimerization inhibitor, in patients with human epidermal growth factor receptor 2-negative metastatic breast cancer. J Clin Oncol 2010;28:1131-7.

19. Gianni L, Dafni U, Gelber RD, et al. Treatment with trastuzumab for 1 year after adjuvant chemotherapy in patients with HER2-positive early breast cancer: a 4-year follow-up of a randomised controlled trial. Lancet Oncol 2011;12:236–244

20. Gianni L, Pienkowski T, Im YH, et al. Efficacy and safety of neoadjuvant pertuzumab and trastuzumab in women with locally advanced, inflammatory, or early HER2-positive breast cancer (NeoSphere): a randomised multicentre, open-label, phase 2 trial. Lancet Oncol 2012;13:25-32.

21. Guarneri V, Frassoldati A, Bottini A, et al. Preoperative chemotherapy plus trastuzumab, lapatinib, or both in human epidermal growth factor receptor 2-positive

operable breast cancer: results of the randomized phase II CHER-LOB study. J Clin Oncol 2012 ;30:1989-95.

22. Holmes FA, Espina VA, Liotta LA, et al. Correlation of clinical and molecular findings with pathologic response to preoperative lapatinib and trastuzumab, separately or in combination, prior to neoadjuvant chemotherapy for HER2 positive breast cancer. Cancer Res 2011;71:Abstract P15-3.

23. Howlader N, Noone AM, Krapcho M, et al. SEER Cancer Statistics Review, 1975 -- 2008, Bethesda, MD: National Cancer Institute. Based on November 2010 SEER data submission, posted to the SEER website, 2011. Available from: http://seer.cancer.gov/csr/ 1975_2008/

24. Ismael G, Hegg R, Muehlbauer S, Heinzmann D, Lum B, Kim SB. Subcutaneous versus intravenous administration of (neo)adjuvant trastuzumab in patients with HER2-positive, clinical stage I-III breast cancer (HannaH study): a phase 3, open-label, multicentre, randomised trial. Lancet Oncol 2012;13:869-78.

25. Jemal A, Bray F, Center MM, et al. Global cancer statistics CA Cancer J Clin 2011;61:69-90

26. Johnston S, Pippen J, Jr., Pivot X, et al. Lapatinib combined with letrozole versus letrozole and placebo as first-line therapy for postmenopausal hormone-receptor-positive metastatic breast cancer. J Clin Oncol 2009;27:5538–46.

27. Kaufman B, Mackey JR, Clemens MR, et al. Trastuzumab plus anastrozole versus anastrozole alone for the treatment of postmenopausal women with human epidermal growth factor receptor 2-positive, hormone receptor-positive metastatic breast cancer: results from the randomized TAnDEM study. J Clin Oncol 2009;27:5529–37.

28. Marty M, Cognetti F, Maraninchi D, et al. Randomized phase II trial of the efficacy and safety of trastuzumab combined with docetaxel in patients with human epidermal growth factor receptor 2-positive metastatic breast cancer administered as first-line treatment: the M77001 study group. J Clin Oncol 2005;23:4265-74.

29. Moja L, Tagliabue L, Balduzzi S, et al. Trastuzumab containing regimens for early breast cancer. Cochrane Database Syst Rev 2012;4:CD006243.

30. Pauletti G, Dandekar S, Rong H, et al. Assessment of methods for tissue-based detection of the HER-2/neu alteration in human breast cancer: a direct comparison of fluorescence in situ hybridization and immunohistochemistry. J Clin Oncol 2000;18:3651-64.

31. Perez EA, Romond EH, Suman VJ, Jet al. Four-year follow-up of trastuzumab plus adjuvant chemotherapy for operable human epidermal growth factor receptor 2-positive breast cancer: joint analysis of data from NCCTG N9831 and NSABP B-31. J Clin Oncol 2011;29:3366–3373

32. Rastogi P, Anderson SJ, Bear HD, et al. Preoperative chemotherapy: updates of National Surgical Adjuvant Breast and Bowel Project Protocols B-18 and B-27. J Clin Oncol 2008;26:778-85.

33. Semiglazov VF, Semiglazov VV, Dashyan GA, Ziltsova EK, Ivanov VG, Bozhok AA. Phase 2 randomized trial of primary endocrine therapy versus chemotherapy in postmenopausal patients with estrogen receptor-positive breast cancer. Cancer 2007;110:244-54

34. Smith IE, Dowsett M, Ebbs SR, et al. Neoadjuvant treatment of postmenopausal breast cancer with anastrozole, tamoxifen, or both in combination: the Immediate Preoperative Anastrozole, Tamoxifen, or Combined with Tamoxifen (IMPACT) multicenter double-blind randomized trial. J Clin Oncol 2005;23:5108-5116

35. Slamon DJ, Leyland-Jones B, Shak S, et al. Use of chemotherapy plus a monoclonal antibody against HER2 for metastatic breast cancer that overexpresses HER2. N Engl J Med 2001;344:783-92.

36. Tan-Chiu E, Yothers G, Romond E, et al. Assessment of cardiac dysfunction in a randomized trial comparing doxorubicin and cyclophosphamide followed by paclitaxel, with or without trastuzumab as adjuvant therapy in node-positive, human epidermal growth factor receptor 2-overexpressing breast cancer: NSABP B-31. J Clin Oncol 2005;23:7811-9.

37. Thurlimann BJ, Keshaviah A, Mouridsen H, et al. BIG 1-98: Randomized doubleblind phase III study to evaluate letrozole (L) vs. tamoxifen (T) as adjuvant endocrine therapy for postmenopausal women with receptor-positive breast cancer. Proc Am Soc Clin Oncol 2005;24:abs 511.

38. Untch M, Loibl S, Bischoff J, et al. Lapatinib versus trastuzumab in combination with neoadjuvant anthracycline-taxane-based chemotherapy (Gepar-Quinto, GBG 44): a randomised phase 3 trial. Lancet Oncol 2012;13: 135–144

39. Wynne C, Harvey V, Schwabe C, Waaka D, McIntyre C, Bittner B. Comparison of subcutaneous and intravenous administration of trastuzumab: a phase I/Ib trial in healthy male volunteers and patients with HER2-positive breast cancer. J Clin Pharmacol 2012 http://dx.doi.org/10.1177/0091270012436560 published online Feb 22.
