# Supplementary figures and images for: HER2DX genomic test in HER2-positive/hormone receptor-positive breast cancer treated with neoadjuvant trastuzumab and pertuzumab: A correlative analysis from the PerELISA trial
Source: eBioMedicine. 2022 Oct 29;85:104320. doi: 10.1016/j.ebiom.2022.104320 (PMC9626543; doi:10.1016/j.ebiom.2022.104320)

# Supplementary Figure 1. PerELISA study design

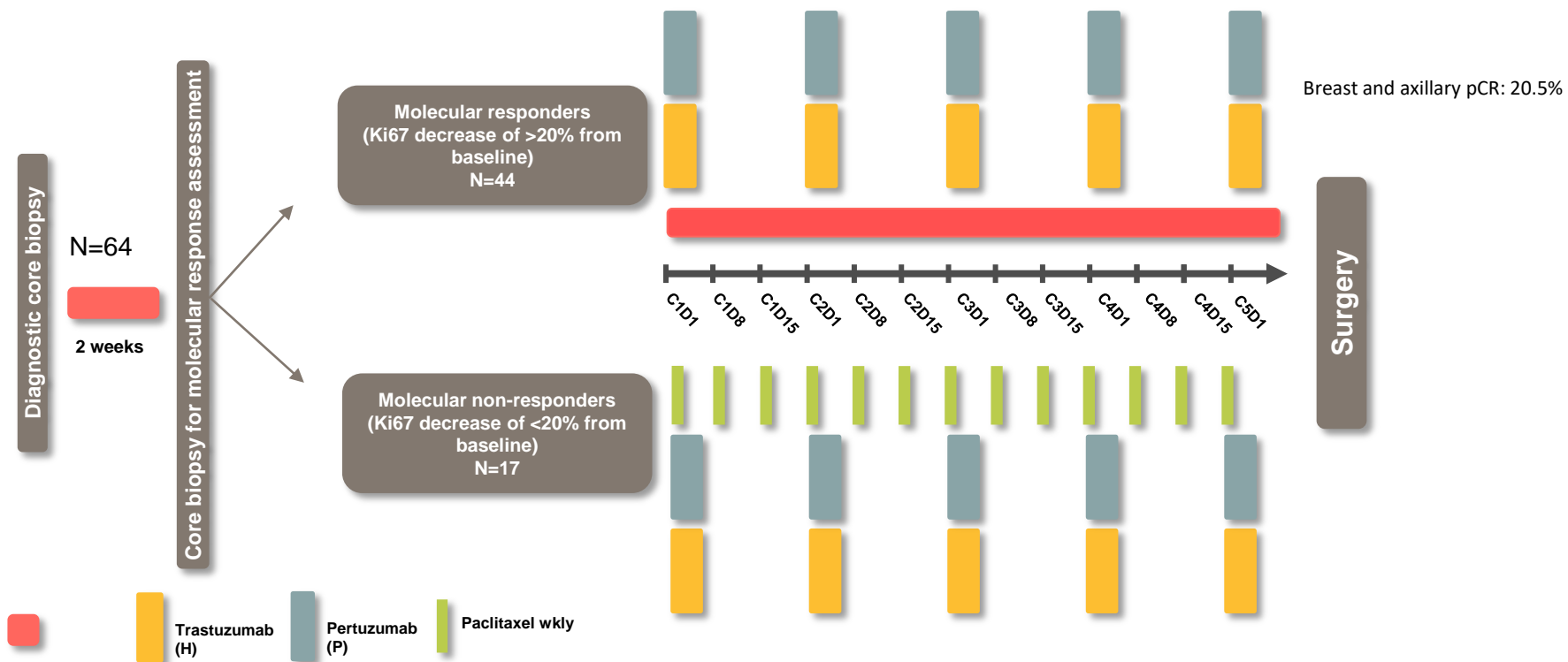

Supplement: Supplementary Figure S1 [file mmc2.pdf]
